# Supplementary material for: Magneto-active elastic shells with tunable buckling strength
Source: Nat Commun. 2021 May 14;12:2831. doi: 10.1038/s41467-021-22776-y (PMC8121925; doi:10.1038/s41467-021-22776-y)
Supplement: Supplementary file 1 — Supplementary Information [file 41467_2021_22776_MOESM1_ESM.pdf]

SUPPLEMENTARY INFORMATION
Magneto-active elastic shells with tunable buckling strength

Dong Yan[1], Matteo Pezzulla[1], Lilian Cruveiller[1,2], Arefeh Abbasi[1], and Pedro M. Reis[1*]

[1]Flexible Structures Laboratory, Institute of Mechanical Engineering, École Polytechnique Fédérale de Lausanne (EPFL), Switzerland

[2]École Polytechnique, Palaiseau, France
[*]Corresponding author: pedro.reis@epfl.ch

Supplementary Note 1 Experimental Protocols

In this note, we elaborate on the protocols that we have developed to fabricate and characterize our hard-magnetic shells. In Section 1.1, we present the coating technique used to fabricate the shell specimens. Then, in Section 1.2, we discuss the methodology followed to characterize the geometry of the engineered defects of the fabricated shells.

1.1 Fabrication protocol

We fabricated magnetic shell specimens using a customized coating protocol (see schematic in Supplementary Fig. 1) that was inspired from our previous work[1,2], while also including some important modifications. The protocol contains 3 steps – (i) Fabrication of the mold, (ii) Fabrication of the shell, and (iii) Magnetization of the shell – that are detailed next.

(i) Fabrication of the mold: An elastic mold was manufactured as the negative of a rigid hemisphere (radius 25.4 mm, stainless steel ball, TIS-GmbH) by pouring liquid VPS solution (base to catalyst ratio of 1:1 in weight, fully mixed and degassed) into a boxed-shaped container with the hemisphere placed on its bottom side (Supplementary Fig. 1**a**). A cylindrical pillar (radius 4.41 mm) was 3D-printed and positioned at a distance of ≈ 0.5 mm above the north pole of the steel ball, in order to produce a circular thin region in the mold. After curing of the VPS, the mold was detached from both the ball and the pillar (Supplementary Fig. 1**b**). Then, we activated the surface of the mold using air plasma generated by a plasma cleaner (PDC-002-HPCE, Harrick Plasma) for 2 minutes and treated it with TMCS (Trimethylchlorosilane, Sigma-Aldrich) in a vacuum chamber for 1 hour. This treatment reduced the adhesion strength between the mold and the MRE to facilitate the demolding of shell specimens during step (ii).

(ii) Fabrication of the shell: The prepared mixture of MRE was poured into the negative spherical mold produced during step (i); see Supplementary Fig. 1**c** and the Methods section of the main article. The pouring was done after waiting for a set time, counting from the completion of mixture preparation (100 s for MRE-22 and 20 s for MRE-32). The purpose of this waiting time was to increase the viscosity to a desired value[2]. With the spherical surface facing down, the gravity-driven viscous flow yielded a thin layer of MRE on the mold (Supplementary Fig. 1**d**). Before the MRE was fully cured, at about 13 min after pouring, we applied constant negative pressure to the mold by extracting the air inside using a syringe pump (Supplementary Fig. 1**e**). Under this depressurization, the soft spot (thin region) of the mold deformed, thereby producing an axisymmetric geometric imperfection in the shell at the pole. This defect was frozen in the shell after curing. During this depressurization stage, the bulk regions of the mold (other than the soft spot) exhibited negligible deformation. Shells made of MRE-22 or MRE-32 were fabricated using molds made out of the corresponding VPS polymer.

(iii) Magnetization of the shell: After fabrication, the shells were then magnetized permanently by saturating the NdPrFeB particles in the MRE using an applied uniaxial magnetic field (4.4 T, perpendicular to the equatorial plane, Supplementary Fig. 1**f**) generated by an impulse magnetizer (IM-K-010020-A, Magnet-Physik Dr. Steingroever GmbH). To constrain the shell in its natural undeformed configuration, the process was conducted before demolding by placing the cured shell (still adhered to the pressurized mold) inside the magnetization coil (MF-AsA-ϕ70×120mm, Magnet-Physik Dr. Steingroever GmbH). We then released the pressure loading and injected VPS mixture into the shell from its bottom to make a

thick band (≈ 6 mm in thickness, Supplementary Fig. 1**g**). This latter step ensured clamped boundary condition at the equator during buckling tests. Finally, the magnetic hemispherical shell containing a geometric defect was peeled off from the mold (Supplementary Fig. 1**h**).

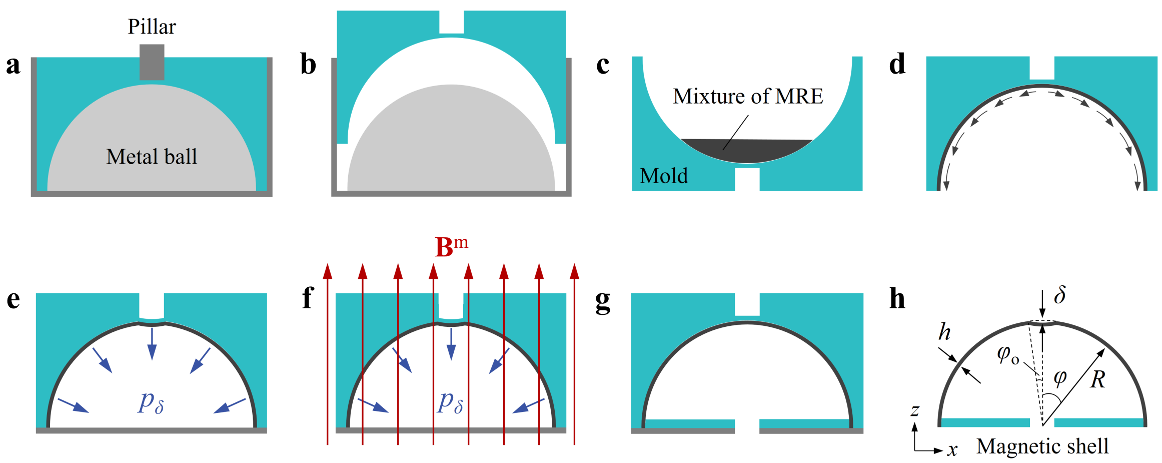

Supplementary Fig. 1: Schematic diagram of the fabrication protocol for precisely imperfect magnetic shells. a, Fabrication of a negative spherical mold containing a soft spot (thin region) at its pole. **b**, Unmolding from the metal ball. **c**, The mold is filled with the liquid mixture of MRE. **d**, The mold is turned upside-down, which leads to a gravity-driven flow of the MRE that generates a thin coating on its surface. **e**, The soft spot (circular, thin, plate-like region) of the mold deforms under the applied negative pressure, p_δ, to produce the geometric imperfection in the shell. **f**, The cured shell, while still adhered to the pressurized mold, is magnetized permanently using an applied magnetic field. **g**, A thick base is made at the equator by injection of additional VPS polymer from the base of the mold. **h**, The final magnetic shell containing a geometric defect is peeled off from the mold. The relevant geometric quantities are shown.

Systematic variation of the defect geometry. The fabrication protocol presented above allows us to independently vary both the defect width (by changing the radius of the pillar used in the mold manufacture; Supplementary Fig. 1**a**) and the defect amplitude (by adjusting the pressure imposed on the mold; Supplementary Fig. 1**e**). For the fabrication of the MRE-22 shells, we applied pressure in the range of $1 \leq p_\delta \leq 9.75\,\text{kPa}$, which yielded defect amplitudes ranging from $\overline{\delta} = 0.14$ to $\overline{\delta} = 3.2$ (Supplementary Fig. 2).

Throughout the study, the half angular width of the defects was fixed at $\varphi_\circ = 11.7 \pm 0.1\,°$ by setting the radius of the soft spot of the mold at 4.41 mm (corresponding to the target half angular width of 10.0 ° set by the radius of the pillar). The minor difference between the obtained value for half angular width and the targeted value can be attributed to small deformations during fabrication near the boundary of the spot, which was not ideally clamped by the surrounding soft bulk regions of the mold. For the MRE-32 shells, we varied the defect amplitude in the range $0.17 \leq \overline{\delta} \leq 3.4$ by changing the pressure in the range $2 \leq p_\delta \leq 13\,\text{kPa}$, with a fixed half angular width of $\varphi_\circ = 11.6 \pm 0.1\,°$. An empirical linear relationship was found between the defect amplitude and the applied pressure (the slope depends on the bending stiffness of the thin region of the mold). These results serve as a convenient set of design guidelines to fabricate the specimens.

1.2 Characterization of the defect geometry

Measurement of the defect profile. To characterize the defect geometry of each shell specimen, the 3D profile of the shell (outer surface) was measured using an optical profilometer (VR-3200, Keyence Corporation). A perfect spherical surface was fitted to the measured data in the region away from the defect located at the pole of the shell. The defect profile, w_δ, was defined as the radial distance between the fitting spherical surface and the measured profile. Due to the axisymmetry of the shell, the 3D profile was averaged latitude-wise to obtain the 2D profile.

A simplified plate model to describe the generated defect geometry. We used a simple model to provide an analytical description of the geometry of the defect. Note that, during pressurization of the mold, the bulk region outside of the soft spot exhibits negligible deformation compared to the soft spot. Moreover, the spherical cap of this soft spot is sufficiently small (with respect to the radius of curvature of the shell), such that it can be regarded as a (nearly) flat plate. Hence, we isolate this thin region and model it as a boundary-clamped circular flat plate loaded by a uniform pressure. The defect profile is determined by the deflection of this plate under the pressure loading. Although the boundary of the thin region was constrained by the flexible bulk

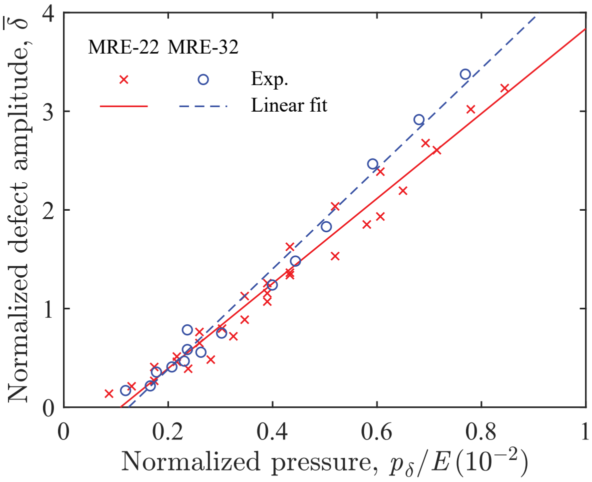

Supplementary Fig. 2: Defect amplitude normalized by the shell thickness, $\bar{\delta} = \delta/h$, versus pressure normalized by the Young's modulus of MRE, p_δ/E, applied during the fabrication of MRE-22 and MRE-32 shells. Half angular width of the defects was fixed at $\varphi_\circ = 11.7\,^\circ$ for MRE-22 shells and at $\varphi_\circ = 11.6\,^\circ$ for MRE-32 shells. The radius of all shells is $R = 25.4\,$mm. The thicknesses of MRE-22 shells and MRE-32 shells are $h = 321.2\,\mu$m and $h = 278.2\,\mu$m, respectively. The experimental data are represented by the symbols and the linear fit by the solid (MRE-22) and dashed (MRE-32) lines.

region, we found that the measured profiles can be well described by the solution corresponding to a clamped boundary condition (see Fig. 2**b** of the main article). However, the amplitude and width of the defect have to be determined through fitting (see details below).

The governing equation can be obtained from classic Kirchhoff-Love theory for an isotropic and homogeneous plate under pure bending[3],

$$\frac{\partial^4 w_\delta}{\partial x^4} + 2\frac{\partial^4 w_\delta}{\partial x^2 \partial y^2} + \frac{\partial^4 w_\delta}{\partial y^4} = 0\,, \tag{1}$$

where w_δ is the out-of-plane displacement of the plate, and x,y are the two in-plane coordinates, respectively. Solving Supplementary Eq. (1) for the specific case of a circular plate of radius, $r_\circ$, and thickness, t, under uniform pressure loading, p_δ, and clamped boundary conditions, yields the following expression for the plate deflection[3]

$$w_\delta(r) = -\frac{p_\delta r_\circ^4}{64D}\left(1 - \frac{r^2}{r_\circ^2}\right)^2\,, \tag{2}$$

where $D = Et^3/12\left(1-\nu^2\right)$ is the bending modulus of the plate, and $r = \sqrt{x^2+y^2}$ is the radial coordinate. Note that we are using t to designate the thickness of the circular plate, so as not to confuse it with the thickness of shells, h, used elsewhere in the study. Recognizing the maximum deflection at the center of the circular plate, $w_\delta(0) = -p_\delta r_\circ^4/(64D)$ as the defect amplitude δ, and $r/r_\circ$ as $\approx \varphi/\varphi_\circ$ (from geometry), the profile of the dimple-like defect, for $\varphi \leq \varphi_\circ$, can be then written as

$$w_\delta(\varphi) = -\delta\left(1 - \frac{\varphi^2}{\varphi_\circ^2}\right)^2\,, \tag{3}$$

where $\varphi_\circ$ is the half angular width of the defect, and φ is the angular distance (polar angle) measured from the center of the defect. Outside of the defect ($\varphi > \varphi_\circ$), we have $w_\delta = 0$.

We determined the amplitude, δ, and half angular width, $\varphi_\circ$, of the defect by fitting Supplementary Eq. (3) to the experimentally measured defect profiles, finding excellent agreement between the two (see Fig. 2**b** of the main article). From Supplementary Eq. (2), we also note that $\delta = w_\delta(0)$ scales linearly with p_δ, which allows us to vary the defect amplitude δ during shell fabrication by adjusting the pressure applied to the mold. In Supplementary Fig. 2, we present the values of δ measured after demolding versus p_δ for a series of MRE-22 and MRE-32 shells, confirming this linear relationship. The slope depends on the bending stiffness of the thin region of the mold. The non-zero intercept on the horizontal axis is due to the natural deflection of the thin region, which might be induced by the pre-stresses developed in the mold during the curing of VPS polymer.

So far, we have provided a simplified theoretical model to aid in the characterization of the profile of the defects in our imperfect shells. This analysis provides an excellent descriptor for the functional form of the defect geometry, even if the underlying two parameters (amplitude and width of the defect) are determined through fitting. In addition to aiding in setting the design parameters for fabrication, the geometric profile of the imperfect shells provided by this model is used directly as an input for the shell model (Supplementary

Note 3). In Supplementary Note 4, we will investigate how the geometric profile affects the onset of buckling by independently varying the underlying geometric parameters in the numerics.

Supplementary Note 2 Experiments

In Section 2.1, we provide information on the generation of the magnetic field in our experiments, including the coils setup with the simulations and experiments performed to characterize the generated field. Then, in Section 2.2, we detail the apparatus and procedure for the buckling tests, with a focus on the generation of the magnetic field.

2.1 Generation and characterization of the magnetic field

Description of the Helmholtz coils used to generate the magnetic field. Two customized multi-turn circular coils with a square cross-section were set up in the Helmholtz configuration to generate a uniaxial uniform magnetic field in their central region. Each coil was manufactured by winding an aluminum circular spool with an insulated magnet wire: 18 turns in the axial direction and 19 turns in the radial direction (Repelec Moteurs S.A.). The magnet wire (enamelled wire, Isomet AG) had a circular cross-section of diameter 1 mm for the copper core and thickness 0.094 mm for the outer insulation layer. The final dimensions of the coil were: 72 mm in inner diameter, 114 mm in outer diameter, and 21 mm in height. The two identical coils were set concentrically, spaced with an axial centre-to-centre distance of 46.5 mm. They were connected in series and powered by a DC power supply that provided a maximum current/power of 25 A/1.5 kW (EA-z 9200-25 T, EA-Elektro-Automatik GmbH). The strength of magnetic field was varied by adjusting the current output from the power supply.

Numerical simulation of the generated magnetic field. We used the commercial package COMSOL Multiphysics (v5.2, COMSOL Inc.) to simulate and characterize the magnetic field generated by the coils described above. These simulations were conducted using the *Magnetic Fields* interface embedded in COMSOL, based on Ampère's Law. Given the axisymmetry of the system, we modeled the 2D cross-section of the two coils, encompassed by a circular region of air (relative permeability 1) that was significantly larger than the size of the system (radius ten times larger than that of the coils). In this setting, we only consider the copper parts (relative permeability 0.999994) with current flowing through; i.e., the aluminum housing of the wires, with a relative permeability of ≈ 1, were neglected. Since the strength of the magnetic field is linearly proportional to the current, I, we normalized the simulations by setting $I = 1$ A.

Experimental characterization of the generated magnetic field. Experimental measurements of the field strength were carried out to characterize the field generated in the experiments, as well as to validate the simulations. An axial Hall probe (HS-AGB5-4805, Magnet-Physik Dr. Steingroever GmbH) was moved along the central axis (vertical) of the two coils (inset of Supplementary Fig. 3**a**), and the corresponding flux density was measured by a Teslameter (FH 55, Magnet-Physik Dr. Steingroever GmbH). Similarly, a transverse Hall probe (HS-TGB5-104010, Magnet-Physik Dr. Steingroever GmbH) was used to measure the flux density along the radial (horizontal) direction (inset of Supplementary Fig. 3**b**). These measurements agree well with the simulation results, along both the axial and radial directions, as shown in Supplementary Fig. 3.

2.2 Buckling tests under combined magnetic and pressure loading

Experimental apparatus for the buckling tests. We measured the critical buckling load of the fabricated magnetic shells under pressure and magnetic loadings by positioning the shell in between the Helmholtz coils, with its pole located in the central region of the field (see Fig. 1**a**,**b** of the main article). The pneumatic loading system comprised a syringe pump (NE-1000, New Era Pump Systems Inc.) and a differential pressure sensor (total error band ±3.7 Pa, 785-HSCDRRN002NDAA5, Honeywell International Inc.), which were both connected to the shell.

Protocol of the buckling test. For each experimental run, under a given external magnetic flux density, the syringe pump was set to extract the air inside the system at a constant flow rate of 0.4 mL/min, so as to depressurize the shell until it buckled. During depressurization, the pressure differential between the outside (atmospheric pressure) and inside of the shell was monitored by the pressure sensor at an acquisition rate of 8 Hz. For each tested shell, we obtained the evolution of pressure loading as a function of the imposed volume extracted by the syringe. The critical buckling pressure is defined as the peak value of this signal, representing the load-carrying capacity of the shell.

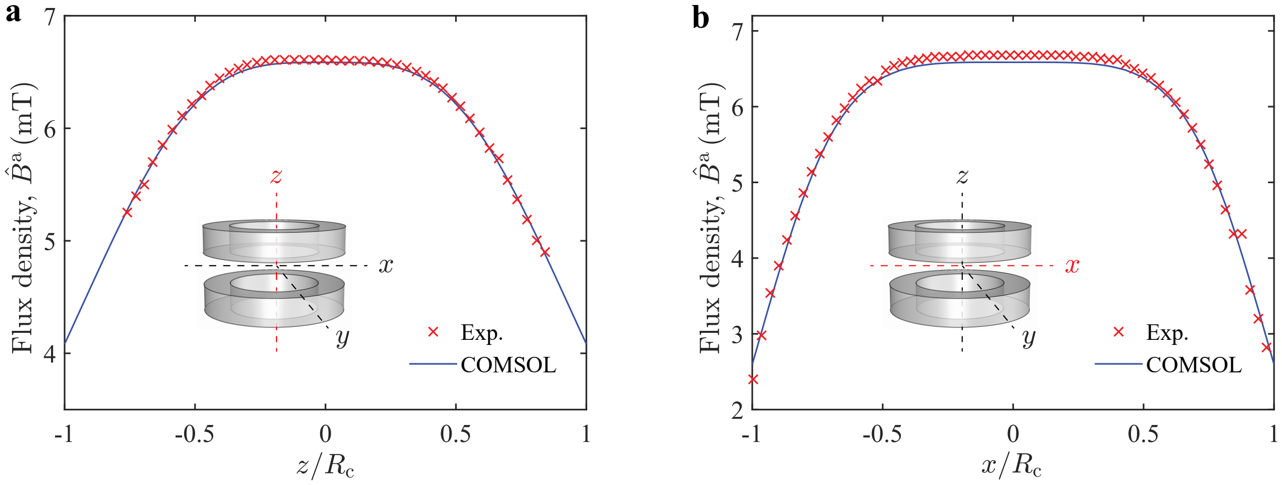

Supplementary Fig. 3: Comparison between the experimentally measured and the computed flux density of the generated magnetic field. The experimental data (symbols) and simulation results obtained using COMSOL (solid lines) along the axial centre line ($x = y = 0$) and the radial centre line ($y = z = 0$) are presented in the plots **a** and **b**, respectively. A current of 1 A is applied to the coils. The radius of the coils is $R_{\rm c}$.

Avoiding overheating of the coils (due to Joule heating) during the experimental tests was crucial. Otherwise, the decrease of inner pressure of the shell p_i (by expanding the air volume V_i using the syringe pump) would be compromised by the increasing temperature T_i of the heated air inside the shell, according to the ideal ideal gas law $p_i = n_i R_i T_i / V_i$ (n_i is the constant number of air molecules in the system and R_i is the gas constant). As a result of overheating, the pressure loading applied on the shell would have decreased before reaching the critical value. This issue was particularly important for the stronger shells (at higher values of $B^{\rm a}$). Noting that the flow rate of 0.4 mL/min for the pressurization was set constant for all experiments, these experiments could run for up to 70 s, which was far too long for the coils to be switched on. This increased running time for some of these runs meant that applying the strong magnetic field continuously throughout the full duration of the test was not feasible. Fortunately, auxiliary experiments demonstrated that the measured buckling pressure under the presence of the magnetic field was insensitive to when the field was switched on along the loading path, as long as this was done prior to buckling. Hence, to obtain reliable results, the field could be switched off from the start of the experimental test and only switched on in the proximity of $p_{\rm max}$, prior to reaching this value. To do so, we considered the cases of $B^{\rm a} > 0$ (stronger shells with respect to no-field) and $B^{\rm a} < 0$ (weaker shells with respect to no-field) differently. When the shell is strengthened by the magnetic field (tests for external flux density $B^{\rm a} > 0$ in Figs. 2**c** and 3 of the main article), buckling occurs at increasing pressure levels with $B^{\rm a}$. To avoid overheating of the coils, the magnetic field was only applied when the pressure reached the level that was 10 Pa below the critical buckling pressure of the non-magnetic case ($B^{\rm a} = 0$). On the other hand, for shells weakened by the magnetic field (in the range $-66 < B^{\rm a}\,[\rm mT] < 0$ in Figs. 2**c** and 3 of the main article), we followed a slightly different procedure while still attempting to minimize overheating. Specifically, we first measured the critical pressure for the lowest value considered for the applied magnetic field ($B^{\rm a} = -66$ mT), corresponding to the weakest conditions (lowest buckling pressure), while switching on the field at the start of the test. For subsequent experiments, we systematically increased the applied field in steps of 6 mT, over the range $-66 < B^{\rm a}\,[\rm mT] < 0$ (11 experiments in total). For the $i^{\rm th}$ experiment, we had the field switched off until the pressure reached the value of $(p_{\rm max}(i-1) - 5)$ [Pa], where $p_{\rm max}(i-1)$ was the critical pressure measured in the previous $(i-1)^{\rm th}$ experiment.

Supplementary Note 3 Theoretical Model

This note is dedicated to the theoretical model that we developed to describe the onset of buckling of axisymmetric hard-magnetic shells. First, we describe the kinematics of axisymmetric shells, followed by the dimensional reduction of the underlying Helmholtz free energy (Section 3.1). To gain insight into this nonlinear magnetic energy, we perform an expansion for small displacements and interpret the physical meaning of the relevant terms at the different orders, namely: magnetic pressure, in-plane force, and torque (Section 3.2). A scaling analysis on these terms is presented in Section 3.3 to help rationalize how the knockdown factor changes with the applied magnetic field. In Section 3.4, we examine their role in the load-carrying behavior of magnetic shells. Emphasis is given to the second-order term of the magnetic energy representing the distributed magnetic

torque relevant to the shell rotation, which is found to dominate the critical buckling conditions of the shells.

3.1 Axisymmetric hard-magnetic shell model

Dimensional reduction of the Helmholtz free energy for axisymmetric shells. As a starting point for the derivation of our hard-magnetic shell model, we use the Helmholtz free energy for ideal hard-magnetic soft materials[4,5]. This specialization of the free energy comprises an elastic part related to material deformation (hereon referred to as *elastic energy*) and a magnetic part describing the work to align the residual magnetic moment along the external magnetic field (hereon referred to as *magnetic energy*). Given that the saturated MRE can be assumed to have the permeability of vacuum[6,5], the magnetic potential energy in the reference configuration of volume V can be simplified as the work to align the residual magnetic moment of the material with the external magnetic field. For 3D scale-free materials, this magnetic potential energy is[6,4,5]

$$\mathcal{U}_{\mathrm{m}} = -\int \frac{1}{\mu_0}\mathbf{F}\mathbf{B}^{\mathrm{r}} \cdot \mathbf{B}^{\mathrm{a}} \, \mathrm{d}V \,, \tag{4}$$

where μ_0 is the permeability of vacuum, $\mathbf{F}$ is the deformation gradient tensor, $\mathbf{B}^{\mathrm{r}}$ is the vector of residual magnetic flux density of the material in the reference configuration, and $\mathbf{B}^{\mathrm{a}}$ is the vector of external magnetic flux density.

In this model, we have neglected the energy corresponding to the magnetic interaction between particles embedded in the MRE. To test the validity of this assumption, we performed a specific test of experiments that confirmed these magnetic interactions do not modify the original shell geometry, nor do they affect the knockdown factor. In the experiments, differently from the fabrication protocol described in Supplementary Note 1, we peeled off each of the shells (Supplementary Fig. 1**e**) from the mold before they were magnetized. We then quantified the defect amplitude of each non-magnetized shell and measured their critical buckling pressure. The shells were then magnetized, after which the same measurements were performed (defect amplitude and critical buckling pressure). No external magnetic field was applied during these buckling tests to evaluate the influence of particle interactions alone. In Supplementary Fig. 4, we present the experimental results for a set of eight, across a range of small ($\overline{\delta} < 1$) and large ($\overline{\delta} > 1$) defects. We found that the defect amplitudes (Supplementary Fig. 4**a**) and the corresponding knockdown factors (Supplementary Fig. 4**b**) measured before and after magnetizing were nearly identical. These results indicate that the particle-particle magnetic interactions were too weak to affect the shells' geometry and buckling behavior, presumably due to the low volume concentration and random distribution of the magnetic particles in the polymer matrix of our MRE. Also, before the onset of buckling, different regions in the shell are still distanced sufficiently; thus, minimizing the associated self-interaction. For more complex settings, where particle interactions may play a role, a higher level of sophistication in the modeling approach will be required, beyond the scope of our present study.

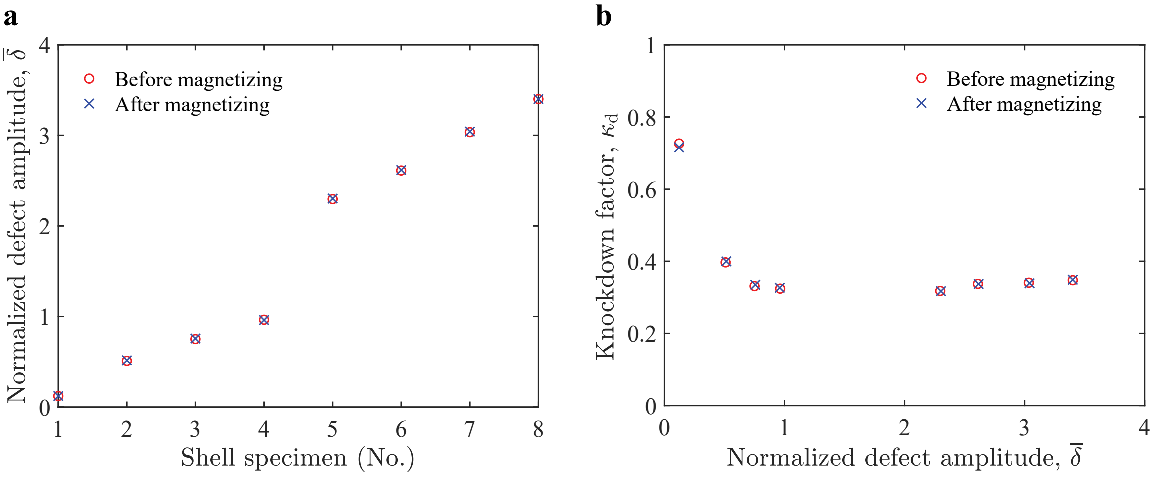

Supplementary Fig. 4: Evaluation of the potential effect of particle-particle magnetic interactions. We tested eight different shell specimens with different degrees of imperfection. **a**, Normalized defect amplitudes, $\overline{\delta}$, and **b**, the corresponding knockdown factors, κ_{d}, of shell specimens before (circles) and after (crosses) they were magnetized. The two sets of measurements are nearly identical, indicating that magnetic interactions are negligible.

In the following, we seek to derive the magnetic energy counterpart for axisymmetric shells by reducing the 3D description of Supplementary Eq. (4) to the 1D profile curve of the middle surface of the shell; a procedure that is commonly referred to as dimensional reduction[7,8,9]. This dimensional reduction will be performed

based on the Kirchhoff-Love assumptions for thin shells [7] (material fibers normal to the middle surface remain normal upon deformation without stretching nor shrinking), within the framework of small strains and large displacements/rotations. We will then follow a variational approach to minimize the total 1D energy via a weak form implementation, similarly to what we have recently proposed for elastic shells subjected to combined pressure and force-indentation loading [10].

3.1.1 Kinematics of axisymmetric shells

Defining the spherical coordinates (φ, θ, η) that represent the polar angle, azimuthal angle, and radial distance, respectively, we parametrize the undeformed reference configuration of the shell as a solid body of revolution

$$\begin{aligned}\mathbf{X}(\varphi,\,\theta,\,\eta) &= \mathring{\mathbf{r}}(\varphi,\,\theta) + \eta\mathring{\mathbf{n}}(\varphi,\,\theta)\\ &= (\mathring{\rho}(\varphi)\cos\theta,\,\mathring{\rho}(\varphi)\sin\theta,\,\mathring{z}(\varphi)) + \eta\mathring{\mathbf{n}}(\varphi,\,\theta)\,,\end{aligned} \tag{5}$$

where $\mathring{\mathbf{r}}(\varphi,\theta) = (\mathring{\rho}(\varphi)\cos\theta,\,\mathring{\rho}(\varphi)\sin\theta,\,\mathring{z}(\varphi))$ represents the undeformed middle surface, with $(\mathring{\rho}, \mathring{z})$ being the 2D Cartesian coordinates of its profile curve, and $\mathring{\mathbf{n}}(\varphi,\theta)$ is the undeformed unit normal vector. The covariant base vectors corresponding to $\mathbf{X}$ can be derived as

$$\begin{aligned}\mathbf{G}_1 &= \frac{\partial\mathbf{X}}{\partial\varphi} = (\mathring{\rho}_{,\varphi}\cos\theta,\,\mathring{\rho}_{,\varphi}\sin\theta,\,\mathring{z}_{,\varphi}) + \eta\mathring{\mathbf{n}}_{,\varphi}\,,\\ \mathbf{G}_2 &= \frac{\partial\mathbf{X}}{\partial\theta} = (-\mathring{\rho}\sin\theta,\,\mathring{\rho}\cos\theta,\,0) + \eta\mathring{\mathbf{n}}_{,\theta}\,,\\ \mathbf{G}_3 &= \frac{\partial\mathbf{X}}{\partial\eta} = \mathring{\mathbf{n}} = \frac{\mathring{\mathbf{r}}_{,\varphi}\times\mathring{\mathbf{r}}_{,\theta}}{|\mathring{\mathbf{r}}_{,\varphi}\times\mathring{\mathbf{r}}_{,\theta}|} = \frac{1}{\sqrt{\mathring{\rho}_{,\varphi}^2+\mathring{z}_{,\varphi}^2}}(-\mathring{z}_{,\varphi}\cos\theta,\,-\mathring{z}_{,\varphi}\sin\theta,\,\mathring{\rho}_{,\varphi})\,.\end{aligned} \tag{6}$$

Under the Kirchhoff-Love kinematic assumptions, the deformed configuration of the shell can be represented by the current middle surface $\mathbf{r}(\varphi,\theta) = (\rho(\varphi)\cos\theta, \rho(\varphi)\sin\theta, z(\varphi))$, together with its normal vector $\mathbf{n}(\varphi,\,\theta)$, as

$$\mathbf{x}(\varphi,\,\theta,\,\rho) = \mathbf{r}(\varphi,\,\theta) + \rho\mathbf{n}(\varphi,\,\theta) = (\rho(\varphi)\cos\theta,\,\rho(\varphi)\sin\theta,\,z(\varphi)) + \eta\mathbf{n}(\varphi,\,\theta)\,. \tag{7}$$

The corresponding covariant base vectors are

$$\begin{aligned}\mathbf{g}_1 &= \frac{\partial\mathbf{x}}{\partial\varphi} = (\rho_{,\varphi}\cos\theta,\,\rho_{,\varphi}\sin\theta,\,z_{,\varphi}) + \eta\mathbf{n}_{,\varphi}\,,\\ \mathbf{g}_2 &= \frac{\partial\mathbf{x}}{\partial\theta} = (-\rho\sin\theta,\,\rho\cos\theta,\,0) + \eta\mathbf{n}_{,\theta}\,,\\ \mathbf{g}_3 &= \frac{\partial\mathbf{x}}{\partial\eta} = \mathbf{n} = \frac{\mathbf{r}_{\varphi}\times\mathbf{r}_{,\theta}}{|\mathbf{r}_{,\varphi}\times\mathbf{r}_{,\theta}|} = \frac{1}{\sqrt{\rho_{,\varphi}^2+z_{,\varphi}^2}}(-z_{,\varphi}\cos\theta,\,-z_{,\varphi}\sin\theta,\,\rho_{,\varphi})\,.\end{aligned} \tag{8}$$

Now, we recall that the deformation gradient is a two-point tensor defined as [11]

$$\mathbf{F} = \mathbf{g}_i\otimes\mathbf{G}^i = G^{ij}\mathbf{g}_i\otimes\mathbf{G}_j\,, \tag{9}$$

where $\mathbf{G}^i$, defined by $\mathbf{G}^i\cdot\mathbf{G}_j = \delta^i_j$ $(i, j = 1, 2, 3)$, are the contravariant base vectors corresponding to $\mathbf{X}$, and G^{ij} are the contravariant components of the metric in the undeformed configuration of the shell. To compute G^{ij}, we first compute the covariant components $G_{ij} = \mathbf{G}_i\cdot\mathbf{G}_j$ as

$$\begin{aligned}G_{11} &= \frac{\left(-\eta\mathring{z}_{,\varphi\varphi}\,\mathring{\rho}_{,\varphi} + \mathring{z}_{,\varphi}\left(\eta\mathring{\rho}_{,\varphi\varphi} + \mathring{z}_{,\varphi}\sqrt{\mathring{z}_{,\varphi}^2+\mathring{\rho}_{,\varphi}^2}\right) + \mathring{\rho}_{,\varphi}^2\sqrt{\mathring{z}_{,\varphi}^2+\mathring{\rho}_{,\varphi}^2}\right)^2}{\left(\mathring{z}_{,\varphi}^2+\mathring{\rho}_{,\varphi}^2\right)^2}\,,\\ G_{22} &= \frac{\left(\eta\mathring{z}_{,\varphi} - \mathring{\rho}\sqrt{\mathring{\rho}_{,\varphi}^2+\mathring{z}_{,\varphi}^2}\right)^2}{\mathring{\rho}_{,\varphi}^2+\mathring{z}_{,\varphi}^2}\,,\\ G_{33} &= 1\,,\\ G_{12} &= 0\,,\\ G_{13} &= 0\,,\\ G_{23} &= 0\,,\end{aligned} \tag{10}$$

resulting in a diagonal metric of the undeformed shell configuration. Hence, the contravariant components can be readily computed as the inverse of the nonzero diagonal covariant components as

$$\begin{aligned}G^{11} &= G_{11}^{-1}\,,\\ G^{22} &= G_{22}^{-1}\,,\\ G^{33} &= 1\,,\end{aligned} \tag{11}$$

with all the other components being zero. With these expressions at hand, we will expand the deformation gradient along the thickness coordinate so as to perform the dimensional reduction by integration of the magnetic energy along the thickness. Before doing so, we first need to briefly introduce the geometry of the middle surface, which will be later required for the development of our theoretical model.

3.1.2 Geometry of the middle surface

We obtain the parametrization of the undeformed middle surface, that is $\mathring{\mathbf{r}}(\varphi,\theta) = (\mathring{\rho}(\varphi)\cos\theta,\ \mathring{\rho}(\varphi)\sin\theta,\ \mathring{z}(\varphi))$ by evaluating the parametrization of the undeformed three-dimensional shell $\mathbf{X}$ for $\eta = 0$. The tangent vectors along the coordinate lines φ and θ (i.e., the covariant base vectors of this surface) can be expressed, respectively, as

$$\begin{aligned} \mathring{\mathbf{a}}_1 &= \frac{\partial \mathring{\mathbf{r}}}{\partial \varphi} = (\mathring{\rho}_{,\varphi}\cos\theta,\ \mathring{\rho}_{,\varphi}\sin\theta,\ \mathring{z}_{,\varphi}) \,, \\ \mathring{\mathbf{a}}_2 &= \frac{\partial \mathring{\mathbf{r}}}{\partial \theta} = (-\mathring{\rho}\sin\theta,\ \mathring{\rho}\cos\theta,\ 0) \,. \end{aligned} \tag{12}$$

The corresponding contravariant base vectors $\mathring{\mathbf{a}}^\alpha$ are defined by $\mathring{\mathbf{a}}^\alpha \cdot \mathring{\mathbf{a}}_\beta = \delta^\alpha_\beta$ $(\alpha,\beta = 1,2)$. By definition[12], the covariant components of the first and second fundamental forms of the undeformed middle surface can be expressed as $\mathring{a}_{\alpha\beta} = \mathring{\mathbf{a}}_\alpha \cdot \mathring{\mathbf{a}}_\beta$ and $\mathring{b}_{\alpha\beta} = -\mathring{\mathbf{n}}_{,\alpha} \cdot \mathring{\mathbf{a}}_\beta$, respectively. Moreover, we denote the square root of the determinant of the covariant metric as $\mathring{a} = \sqrt{\det(\mathring{a}_{\alpha\beta})}$, which will be used in the area measure.

Similarly, if we evaluate the parametrization of the deformed three-dimensional shell, $\mathbf{x}$, at $\eta = 0$, we obtain the parametrization of the deformed middle surface, that is $\mathbf{r}(\varphi,\theta) = (\rho(\varphi)\cos\theta,\ \rho(\varphi)\sin\theta,\ z(\varphi))$. The covariant base vectors can be expressed as

$$\begin{aligned} \mathbf{a}_1 &= \frac{\partial \mathbf{r}}{\partial \varphi} = (\rho_{,\varphi}\cos\theta,\ \rho_{,\varphi}\sin\theta,\ z_{,\varphi}) \,, \\ \mathbf{a}_2 &= \frac{\partial \mathbf{r}}{\partial \theta} = (-\rho\sin\theta,\ \rho\cos\theta,\ 0) \,. \end{aligned} \tag{13}$$

The contravariant base vectors, first and second fundamental forms associated to the deformed middle surface can be computed with the same expressions we showed for the undeformed middle surface, employing the quantities without the accents. Having completed the geometric description of our system, we can now proceed to perform the dimensional reduction of the magnetic energy.

3.1.3 Dimensional reduction of the magnetic energy for axisymmetric shells

We seek to perform a dimensional reduction of the magnetic potential energy for 3D scale-free materials in Supplementary Eq. (4), from 3D to 1D. To do so, consider the specific case for the configuration of the magnetic field in our experiments reported in the main article, where

$$\begin{aligned} \mathbf{B}^{\mathrm{r}} &= B^{\mathrm{r}}\mathbf{e}_3 \,, \\ \mathbf{B}^{\mathrm{a}} &= B^{\mathrm{a}}\mathbf{e}_3 \,. \end{aligned} \tag{14}$$

First of all, the elementary volume can be written as

$$\mathrm{d}V = \mathring{a}(1 - 2\eta\mathring{H} + \eta^2\mathring{K})\,\mathrm{d}\varphi\mathrm{d}\theta\mathrm{d}\eta \,, \tag{15}$$

where we used the expression of the square root of the determinant of the undeformed three-dimensional metric $\mathring{G}$ for a stack of surfaces[7,13], namely $\mathring{G} = \mathring{a}(1 - 2\eta\mathring{H} + \eta^2\mathring{K})$, with $\mathring{H}$ and $\mathring{K}$ denoting the mean and the Gaussian curvatures of the undeformed middle surface, respectively. Then, expressing the deformation gradient by making use of Supplementary Eqs. (14), (10) and (11), we can write the integrand of Supplementary Eq. (4), that is, the density of the magnetic potential, as

$$\begin{aligned} -\frac{1}{\mu_0}\mathbf{F}\mathbf{B}^{\mathrm{r}} \cdot \mathbf{B}^{\mathrm{a}} &= -\frac{1}{\mu_0}B^{\mathrm{r}}B^{\mathrm{a}}\mathbf{F}\mathbf{e}_3 \cdot \mathbf{e}_3 \\ &= -\frac{1}{\mu_0}B^{\mathrm{r}}B^{\mathrm{a}}G^{ij}(\mathbf{g}_i \cdot \mathbf{e}_3)(\mathbf{G}_j \cdot \mathbf{e}_3) \\ &= -\frac{1}{\mu_0}B^{\mathrm{r}}B^{\mathrm{a}}\left(G^{11}(\mathring{z}_{,\varphi} + \eta\mathring{\mathbf{n}}_{,\varphi} \cdot \mathbf{e}_3)(z_{,\varphi} + \eta\mathbf{n}_{,\varphi} \cdot \mathbf{e}_3) + \frac{\mathring{\rho}_{,\varphi}\,\rho_{,\varphi}}{\sqrt{\mathring{\rho}_{,\varphi}^2 + \mathring{z}_{,\varphi}^2}\sqrt{\rho_{,\varphi}^2 + z_{,\varphi}^2}} \right) . \end{aligned} \tag{16}$$

In the next step of the dimensional reduction procedure, we integrate the magnetic energy along the thickness of the reference configuration of the shell, by making use of Supplementary Eqs. (15) and (16). The zeroth order terms in η yield terms that are akin to a stretching energy (i.e., proportional to the thickness). The linear

terms in η vanish upon integration along the thickness direction from $\eta = -h/2$ to $\eta = h/2$. The higher-order terms, $\mathcal{O}(\eta^2)$, would give bending-like contributions to the energy, which we evaluated numerically to be much smaller than the stretching-like contribution, with no effect on the solution. Then, the integrand (Supplementary Eq. (16)) becomes

$$-\frac{1}{\mu_0}\mathbf{F}\mathbf{B}^{\mathrm{r}}\cdot\mathbf{B}^{\mathrm{a}}\,\mathrm{d}V = -\frac{1}{\mu_0}\left(\frac{\mathring{\rho}_{,\varphi}\,\rho_{,\varphi}}{\sqrt{\mathring{\rho}_{,\varphi}^{\,2}+\mathring{z}_{,\varphi}^{\,2}}\sqrt{\rho_{,\varphi}^{\,2}+z_{,\varphi}^{\,2}}}+\frac{\mathring{z}_{,\varphi}\,z_{,\varphi}}{\mathring{\rho}_{,\varphi}^{\,2}+\mathring{z}_{,\varphi}^{\,2}}+\mathcal{O}(\eta^2)\right)\mathring{a}\mathrm{d}\varphi\mathrm{d}\theta\mathrm{d}\eta\,. \tag{17}$$

Finally, we obtain the 1D reduced magnetic energy for an axisymmetric shell

$$\begin{aligned}\overline{\mathcal{U}}_{\mathrm{m}} &= \frac{4(1-\nu^2)}{\pi EhR^2}\int_{-h/2}^{h/2}\int_0^{2\pi}\int_0^{\pi/2}-\frac{1}{\mu_0}\mathbf{F}\mathbf{B}^{\mathrm{r}}\cdot\mathbf{B}^{\mathrm{a}}\;\mathring{a}\mathrm{d}\varphi\mathrm{d}\theta\mathrm{d}\eta\,,\\ &= -\frac{8(1-\nu^2)}{R^2}\frac{B^{\mathrm{r}}B^{\mathrm{a}}}{\mu_0 E}\int_0^{\pi/2}\mathcal{F}(\mathring{\rho}_{,\varphi}\,,\mathring{z}_{,\varphi}\,,\rho_{,\varphi}\,,z_{,\varphi}\,)\;\mathring{a}\mathrm{d}\varphi\,,\end{aligned} \tag{18}$$

which we have normalized by $\pi EhR^2/(4(1-\nu^2))$. For simplicity, we have introduced the following dimensionless function in Supplementary Eq. (18)

$$\mathcal{F} = \frac{\mathring{\rho}_{,\varphi}\,\rho_{,\varphi}}{\sqrt{\mathring{\rho}_{,\varphi}^{\,2}+\mathring{z}_{,\varphi}^{\,2}}\sqrt{\rho_{,\varphi}^{\,2}+z_{,\varphi}^{\,2}}}+\frac{\mathring{z}_{,\varphi}\,z_{,\varphi}}{\mathring{\rho}_{,\varphi}^{\,2}+\mathring{z}_{,\varphi}^{\,2}}\,, \tag{19}$$

which depends on the initial and deformed geometries of the shell. Also, note that $\mathring{a} = \sqrt{\mathring{\rho}^2(\mathring{\rho}_{,\varphi}^{\,2}+\mathring{z}_{,\varphi}^{\,2})}$ is the area measure (i.e., the square root of the determinant of the metric of the undeformed middle surface). A dimensionless *magneto-elastic parameter*,

$$\lambda_{\mathrm{m}} = \frac{B^{\mathrm{r}}B^{\mathrm{a}}}{\mu_0 E}\,, \tag{20}$$

emerges from this analysis, representing the magneto-elastic coupling for thin magnetic shells.

3.1.4 Elastic energy and live pressure potential

So far, we have reduced the dimension of the magnetic potential from 3D to 1D, leaving the elastic energy and the live pressure potential aside as these are already known in the shell literature[7,14,15,10]. Next, we recall and briefly report these energies, while emphasizing the underlying assumptions at their origin.

The elastic energy $\mathcal{U}_{\mathrm{e}}$, as proposed by Koiter[14,7], is valid for small strains since it stems from the Kirchhoff-Saint Venant three-dimensional strain energy. Normalized by $\pi EhR^2/(4(1-\nu^2))$, it reads[10]

$$\overline{\mathcal{U}}_{\mathrm{e}} = \frac{1}{R^2}\int_0^{\pi/2}[(1-\nu)\mathrm{tr}\,(\mathbf{a}-\mathring{\mathbf{a}})^2+\nu\mathrm{tr}^{\,2}(\mathbf{a}-\mathring{\mathbf{a}})]\mathring{a}\,\mathrm{d}\varphi+\frac{1}{3}\left(\frac{h}{R}\right)^2\int_0^{\pi/2}[(1-\nu)\mathrm{tr}\,(\mathbf{b}-\mathring{\mathbf{b}})^2+\nu\mathrm{tr}^{\,2}(\mathbf{b}-\mathring{\mathbf{b}})]\mathring{a}\,\mathrm{d}\varphi\,, \tag{21}$$

where the first term is the (dimensionless) stretching energy, and the second term is the (dimensionless) bending energy. Note that we have introduced the trace operator in the undeformed metric, tr , such that, for example, $\mathrm{tr}\,(\mathbf{a}) = \mathring{a}^{\alpha\beta}a_{\alpha\beta}$.

The live pressure potential, normalized by $\pi EhR^2/(4(1-\nu^2))$, can be expressed as[10]

$$\overline{\mathcal{U}}_{\mathrm{p}} = 8(1-\nu^2)\frac{p}{E}\Delta\overline{V} = 8(1-\nu^2)\frac{1}{3hR^2}\frac{p}{E}\left[\int_0^{\pi/2}\mathbf{r}\cdot\mathbf{n}\,a\mathrm{d}\varphi-\int_0^{\pi/2}\mathring{\mathbf{r}}\cdot\mathring{\mathbf{n}}\,\mathring{a}\mathrm{d}\varphi\right]. \tag{22}$$

Finally, the total dimensionless energy is the sum of the elastic energy, the live pressure potential, and the magnetic energy:

$$\overline{\mathcal{U}} = \overline{\mathcal{U}}_{\mathrm{e}}+\overline{\mathcal{U}}_{\mathrm{p}}+\overline{\mathcal{U}}_{\mathrm{m}}\,. \tag{23}$$

Numerical implementation: In our computer simulations, the equilibrium equations can be obtained by minimizing the total energy $\overline{\mathcal{U}}$ for all possible displacements of the shell. These equilibrium equations are solved via the Newton-Raphson method using the commercial package COMSOL Multiphysics (v. 5.2, COMSOL Inc.). Details of the implementation of our numerical procedure are provided in Ref. [10], albeit without the magnetic contribution derived above.

3.2 Physical interpretation of the reduced magnetic energy

Above, we derived an expression for the reduced magnetic energy, $\overline{\mathcal{U}}_{\mathrm{m}}$ in Supplementary Eq. (18). We now seek to provide a physical interpretation of the various terms involved in $\overline{\mathcal{U}}_{\mathrm{m}}$, focusing in the limit of small displacements. Due to axisymmetry, we express the displacement field of the shell profile in the covariant basis $\{\mathring{\mathbf{a}}_1, \mathring{\mathbf{n}}\}$, introduced before, as

$$\mathbf{u} = \check{\mathbf{u}} + w\mathring{\mathbf{n}} = u^{\alpha}\mathring{\mathbf{a}}_{\alpha} + w\mathring{\mathbf{n}}\,, \tag{24}$$

with $u^2 = 0$, because of axisymmetric deformations. Notice that u^{α} are the contravariant components of the in-plane displacement field (along the covariant base vectors $\mathring{a}_{\alpha}$ defined above), whereas w is the normal displacement[7]. Then, since $\mathbf{r} = \mathring{\mathbf{r}} + \mathbf{u}$, we can expand the integrand of Supplementary Eq. (18) up to second order in the displacement field to find

$$\begin{aligned}\overline{\mathcal{U}}_{\mathrm{m}} = -\frac{8(1-\nu^2)}{R^2}\lambda_{\mathrm{m}}\int_0^{\pi/2}\Big(1 + \frac{\mathring{z},_{\varphi}^2}{(\mathring{\rho},_{\varphi}^2 + \mathring{z},_{\varphi}^2)^2}\mathring{\mathbf{a}}_1\cdot\mathbf{u},_1 - \frac{\mathring{\rho},_{\varphi}^2}{2(\mathring{\rho},_{\varphi}^2 + \mathring{z},_{\varphi}^2)^2}\left(\mathring{\mathbf{n}}\cdot\mathbf{u},_1\right)^2 \\ + \frac{\mathring{\rho},_{\varphi}\mathring{z},_{\varphi}}{(\mathring{\rho},_{\varphi}^2 + \mathring{z},_{\varphi}^2)^{5/2}}\left(\mathring{\mathbf{n}}\cdot\mathbf{u},_1\right)\left(\mathring{\mathbf{a}}_1\cdot\mathbf{u},_1\right)\Big)\,\mathring{a}\mathrm{d}\varphi + \mathcal{O}(|\mathbf{u}|^3)\,,\end{aligned} \tag{25}$$

where the constant term, which does not depend on the displacement field, will be neglected from hereon.

We will now analyze each term in Supplementary Eq. (25), separately, with the goal of understanding their physical significance. Specifically, in the subsequent sub-sections, we will arrive at the following findings

(i) **The linear term** proportional to $\mathring{\mathbf{a}}_1\cdot\mathbf{u},_1$, defined as

$$\overline{\mathcal{U}}_{\mathrm{m}}^{(1)} = -\frac{8(1-\nu^2)}{R^2}\lambda_{\mathrm{m}}\int_0^{\pi/2}\frac{\mathring{z},_{\varphi}^2}{(\mathring{\rho},_{\varphi}^2 + \mathring{z},_{\varphi}^2)^2}\mathring{\mathbf{a}}_1\cdot\mathbf{u},_1\,\mathring{a}\mathrm{d}\varphi\,, \tag{26}$$

represents the combined action of distributed tangential and normal (dead pressure-like) forces;

(ii) **The second-order term** proportional to $\left(\mathring{\mathbf{n}}\cdot\mathbf{u},_1\right)^2$, defined as

$$\overline{\mathcal{U}}_{\mathrm{m}}^{(2)\mathrm{A}} = \frac{8(1-\nu^2)}{R^2}\lambda_{\mathrm{m}}\int_0^{\pi/2}\frac{\mathring{\rho},_{\varphi}^2}{2(\mathring{\rho},_{\varphi}^2 + \mathring{z},_{\varphi}^2)^2}\left(\mathring{\mathbf{n}}\cdot\mathbf{u},_1\right)^2\,\mathring{a}\mathrm{d}\varphi\,, \tag{27}$$

is equivalent to a distribution of linear rotational springs, and dominates over all the other terms;

(iii) **The second-order term** proportional to $\left(\mathring{\mathbf{n}}\cdot\mathbf{u},_1\right)\left(\mathring{\mathbf{a}}_1\cdot\mathbf{u},_1\right)$, which we define as

$$\overline{\mathcal{U}}_{\mathrm{m}}^{(2)\mathrm{B}} = -\frac{8(1-\nu^2)}{R^2}\lambda_{\mathrm{m}}\int_0^{\pi/2}\frac{\mathring{\rho},_{\varphi}\mathring{z},_{\varphi}}{(\mathring{\rho},_{\varphi}^2 + \mathring{z},_{\varphi}^2)^{5/2}}\left(\mathring{\mathbf{n}}\cdot\mathbf{u},_1\right)\left(\mathring{\mathbf{a}}_1\cdot\mathbf{u},_1\right)\,\mathring{a}\mathrm{d}\varphi\,, \tag{28}$$

represents a distribution of torques that are linear functions of the displacement field.

3.2.1 First-order term in Supplementary Eq. (25)

We start by considering the first-order term in Supplementary Eq. (25), defined as $\overline{\mathcal{U}}_{\mathrm{m}}^{(1)}$ in Supplementary Eq. (26), which is proportional to $\mathring{\mathbf{a}}_1\cdot\mathbf{u},_1$. In words, this term represents the component of the longitudinal derivative of the displacement vector along the first covariant base vector (again longitude). Note that, using the covariant basis, we can establish the following identity

$$\mathring{\mathbf{a}}_1\cdot\mathbf{u},_1 = \mathring{\mathbf{a}}_1\cdot[\check{\mathbf{u}},_1 + (w\mathring{\mathbf{n}}),_1] = \mathring{\mathbf{a}}_1\cdot[\check{\mathbf{u}},_1 + w,_1\mathring{\mathbf{n}} - w\mathring{b}_1^1\mathring{\mathbf{a}}_1] = \mathring{\mathbf{a}}_1\cdot\check{\mathbf{u}},_1 - \mathring{b}_{11}w\,, \tag{29}$$

where we used the Weingarten formulae[12] to express $\mathring{\mathbf{n}},_1 = -\mathring{b}_1^1\mathring{\mathbf{a}}_1$, with $\mathring{b}_1^1$ denoting the mixed component along the latitudinal coordinate of the second fundamental form of the undeformed middle surface. We already considered the orthogonality of the spherical coordinates and the fact that the coordinate lines are principal. Then, the approximation for small displacements of the first-order term of the magnetic energy can be written as

$$\overline{\mathcal{U}}_{\mathrm{m}}^{(1)} = -\frac{8(1-\nu^2)}{R^2}\lambda_{\mathrm{m}}\int_0^{\pi/2}\mathring{\psi}(\varphi)\Big(\frac{\mathring{\mathbf{a}}_1\cdot\check{\mathbf{u}},_1 - \mathring{b}_{11}w}{R^2}\Big)\,\mathring{a}\mathrm{d}\varphi = \overline{\mathcal{U}}_{\mathrm{m}}^{(1u)} + \overline{\mathcal{U}}_{\mathrm{m}}^{(1w)}\,, \tag{30}$$

where we highlighted the two different parts of this term proportional to the in-plane displacement and the normal displacement, respectively, and where

$$\mathring{\psi}(\varphi) = R^2\frac{\mathring{z},_{\varphi}^2}{(\mathring{\rho},_{\varphi}^2 + \mathring{z},_{\varphi}^2)^2}\,, \tag{31}$$

is a known dimensionless function depending on the undeformed configuration of the shell.

Magnetic pressure. If we compare the term $\overline{\mathcal{U}}_{\mathrm{m}}^{(1w)}$, proportional to the normal displacement, to the potential of a dead pressure, namely $\overline{\mathcal{U}}_{\mathrm{p}}^{\mathrm{dead}} = \frac{8(1-\nu^2)}{R^2}\int_0^{\pi/2} \frac{p}{E}\frac{w}{h}\,\mathring{a}\mathrm{d}\varphi$ (positive pressure for a shell under compression), we can define a dimensionless contribution to the magnetic loading (normalized by E) that is analogous to a dead pressure,

$$\frac{p_{\mathrm{m}}(\varphi)}{E} = \lambda_{\mathrm{m}}\frac{h}{R}\frac{\mathring{b}_{11}}{R}\mathring{\psi}(\varphi)\,. \tag{32}$$

For a perfect spherical shell with $\mathring{\rho} = R\sin\varphi$, $\mathring{z} = R\cos\varphi$, $\mathring{b}_{11} = -R$, and $\mathring{a} = R^2\sin\varphi$, Supplementary Eq. (32) reduces to

$$\frac{p_{\mathrm{m}}(\varphi)}{E} = \lambda_{\mathrm{m}}\frac{h}{R}\sin^2(\varphi)\,. \tag{33}$$

This equivalent magnetic dead pressure varies across the shell, being zero at the north pole. Notice that the magnitude of this normal force is dictated by the magneto-elastic parameter, λ_m, defined in Supplementary Eq. (20), with a spatial variation imposed via $\mathring{\psi}(\varphi)$.

Distributed, tangential magnetic forces. Regarding the term proportional to the in-plane displacement, that is $\overline{\mathcal{U}}_{\mathrm{m}}^{(1u)}$, we rewrite it as

$$\begin{aligned}\overline{\mathcal{U}}_{\mathrm{m}}^{(1u)} &= -\frac{8(1-\nu^2)}{R^2}\lambda_{\mathrm{m}}\int_0^{\pi/2}\mathring{\psi}(\varphi)\frac{\mathring{\mathbf{a}}_1\cdot\check{\mathbf{u}}_{,1}}{R^2}\,\mathring{a}\mathrm{d}\varphi\\ &= -\frac{8(1-\nu^2)}{R^2}\lambda_{\mathrm{m}}\int_0^{\pi/2}\mathring{\psi}(\varphi)\frac{\mathring{\mathbf{a}}_1\cdot\left(u^1_{,1}\mathring{\mathbf{a}}_1 + u^1\mathring{\mathbf{a}}_{1,1}\right)}{R^2}\,\mathring{a}\mathrm{d}\varphi\\ &= -\frac{8(1-\nu^2)}{R^2}\lambda_{\mathrm{m}}\int_0^{\pi/2}\mathring{\psi}(\varphi)\frac{1}{R^2}\left[u_{1,1} + \frac{1}{2}\frac{\mathring{a}_{11,1}}{\mathring{a}_{11}}u_1\right]\mathring{a}\mathrm{d}\varphi\,,\end{aligned} \tag{34}$$

where we have used the Gauss-Weingarten formula[12]

$$\mathring{\mathbf{a}}_{1,1} = \mathring{b}_{11}\mathring{\mathbf{n}} + \nabla_1\mathbf{a}_1 = \mathring{b}_{11}\mathring{\mathbf{n}} + \mathring{\Gamma}^1_{11}\mathring{\mathbf{a}}_1 = \mathring{b}_{11}\mathring{\mathbf{n}} + \frac{1}{2}\frac{\mathring{a}_{11,1}}{\mathring{a}_{11}}\mathring{\mathbf{a}}_1\,, \tag{35}$$

with ∇_1 denoting the covariant derivative along $\mathring{\mathbf{a}}_1$, and $\mathring{\Gamma}^1_{11}$ is a Christoffel symbol of the second kind[12]. Then we integrate Supplementary Eq. (34) by parts to obtain

$$\overline{\mathcal{U}}_{\mathrm{m}}^{(1u)} = -\frac{8(1-\nu^2)}{R^4}\lambda_{\mathrm{m}}\Big\{\mathring{\psi}(\varphi)\mathring{a}u_1\big|_0^{\pi/2} + \int_0^{\pi/2}\Big[-\big(\mathring{\psi}(\varphi)\mathring{a}\big)_{,1}u_1 + \frac{1}{2}\mathring{\psi}(\varphi)\frac{\mathring{a}_{11,1}}{\mathring{a}_{11}}\mathring{a}u_1\Big]\,\mathrm{d}\varphi\Big\}\,. \tag{36}$$

The first addend on the RHS of Supplementary Eq. (36) vanishes because of the clamped boundary condition at the equator ($u_1|_{\varphi=\pi/2} = 0$). Consequently, Supplementary Eq. (36) becomes

$$\overline{\mathcal{U}}_{\mathrm{m}}^{(1u)} = -\frac{8(1-\nu^2)}{R^2}\lambda_{\mathrm{m}}\int_0^{\pi/2}\frac{1}{R^2}\Big(-\frac{(\mathring{\psi}(\varphi)\mathring{a})_{,1}}{\mathring{a}} + \frac{1}{2}\mathring{\psi}(\varphi)\frac{\mathring{a}_{11,1}}{\mathring{a}_{11}}\Big)u_1\,\mathring{a}\mathrm{d}\varphi\,, \tag{37}$$

which we can also write as

$$\overline{\mathcal{U}}_{\mathrm{m}}^{(1u)} = -\frac{8(1-\nu^2)}{R^2}\int_0^{\pi/2}\mathbf{f}_{\mathrm{m}}\cdot\check{\mathbf{u}}\,\mathring{a}\mathrm{d}\varphi\,, \tag{38}$$

where we have defined the distributed tangential forces induced by the magnetic field as

$$\mathbf{f}_{\mathrm{m}} = f^\alpha\mathring{\mathbf{a}}_\alpha = \frac{\lambda_{\mathrm{m}}}{R^2}\Big(-\frac{(\mathring{\psi}(\varphi)\mathring{a})_{,1}}{\mathring{a}} + \frac{1}{2}\mathring{\psi}(\varphi)\frac{\mathring{a}_{11,1}}{\mathring{a}_{11}}\Big)\mathring{\mathbf{a}}_1 = f^1\mathring{\mathbf{a}}_1\,,\quad \text{with}\quad f^2 = 0\,. \tag{39}$$

In the case of a perfect spherical shell, this term is equal to

$$f^1 = -\frac{3}{2}\lambda_{\mathrm{m}}R^{-2}\sin(2\varphi)\,. \tag{40}$$

In conclusion, the first-order contribution to the magnetic energy,

$$\overline{\mathcal{U}}_{\mathrm{m}}^{(1)} = \frac{8(1-\nu^2)}{R^2}\int_0^{\pi/2}\Big(\frac{p_{\mathrm{m}}}{E}\frac{w}{h} - \mathbf{f}_{\mathrm{m}}\cdot\check{\mathbf{u}}\Big)\,\mathring{a}\mathrm{d}\varphi\,, \tag{41}$$

can be regarded as a combination of distributed normal and tangential forces that vary across the shell. We now move forward to analyze the second-order terms of $\overline{\mathcal{U}}_{\mathrm{m}}$.

3.2.2 Second-order terms in Supplementary Eq. (25)

Magnetic torque proportional to the shell rotation. We now turn to the term $\overline{\mathcal{U}}_{\mathrm{m}}^{(2)\mathrm{A}}$, which is the second-order term proportional to $(\mathring{\mathbf{n}}\cdot\mathbf{u}_{,1})^2$. We can rewrite the term $\mathring{\mathbf{n}}\cdot\mathbf{u}_{,1}$ by using the covariant basis as

$$\mathring{\mathbf{n}}\cdot\mathbf{u}_{,1} = \mathring{\mathbf{n}}\cdot\left[(u^\alpha\mathring{\mathbf{a}}_\alpha)_{,1} + (w\mathring{\mathbf{n}})_{,1}\right] = \mathring{\mathbf{n}}\cdot\left[\Big(u^1_{,1} + \frac{1}{2}\frac{\mathring{a}_{11,1}}{\mathring{a}_{11}}u^1 - w\mathring{b}^1_1\Big)\mathring{\mathbf{a}}_1 + (\mathring{b}_{11}u^1 + w_{,1})\mathring{\mathbf{n}}\right] = w_{,1} + \mathring{b}^1_1 u_1\,. \tag{42}$$

The quantity above is the first covariant component of the rotation vector $\mathbf{q} = (\delta\mathring{\mathbf{a}}_\alpha\cdot\mathring{\mathbf{n}})\mathring{\mathbf{a}}^\alpha = -(\delta\mathring{\mathbf{n}}\cdot\mathring{\mathbf{a}}_\alpha)\mathring{\mathbf{a}}^\alpha$, which is defined, for example in Ref.[7,16], such that the rotation of a material fiber defined by the unit vector $\mathbf{t}$ is given by $\mathbf{q}\cdot\mathbf{t}$. The rotation vector for small displacement can be expressed as[7,16]

$$\mathbf{q} = (w_{,\alpha} + \mathring{b}^\beta_\alpha u_\beta)\mathring{\mathbf{a}}^\alpha\,, \tag{43}$$

where $\mathring{b}^\beta_\alpha$ is the mixed components of the second fundamental form of the undeformed middle surface. In the case of axisymmetry, we have

$$\mathbf{q} = q_1\mathring{\mathbf{a}}^1 = (w_{,1} + \mathring{b}^1_1 u_1)\mathring{\mathbf{a}}^1\,, \tag{44}$$

with $q_2 = 0$. Therefore, we can write

$$\overline{\mathcal{U}}_{\mathrm{m}}^{(2)\mathrm{A}} = \frac{8(1-\nu^2)}{R^2}\lambda_{\mathrm{m}}\int_0^{\pi/2}\frac{1}{2}\mathring{\chi}(\varphi)\mathbf{q}\cdot\mathbf{q}\,\mathring{a}\mathrm{d}\varphi = \frac{8(1-\nu^2)}{R^2}\int_0^{\pi/2}\frac{1}{2}k\mathbf{q}\cdot\mathbf{q}\,\mathring{a}\mathrm{d}\varphi\,, \tag{45}$$

where

$$k = \lambda_{\mathrm{m}}\mathring{\chi}(\varphi)\,, \tag{46}$$

and

$$\mathring{\chi}(\varphi) = \frac{\mathring{\rho}_{,\varphi}^{\,2}}{\mathring{\rho}_{,\varphi}^{\,2} + \mathring{z}_{,\varphi}^{\,2}} \tag{47}$$

is a dimensionless geometric function depending exclusively on the undeformed configuration of the shell, similarly to $\mathring{\psi}(\varphi)$. In conclusion, the term $\overline{\mathcal{U}}_{\mathrm{m}}^{(2)\mathrm{A}}$ can be interpreted as the energy potential of a distribution of linear torques $\boldsymbol{\tau}$ along the shell, defined as

$$\boldsymbol{\tau} = -k\mathbf{q}\,, \tag{48}$$

where k, defined in Supplementary Eq. (46), is the equivalent rotational stiffness. For a perfect hemispherical shell, k is maximum at the pole and decreases monotonically to zero at the equator.

Magnetic torque proportional to the in-plane displacement. Finally, we turn into the second-order term proportional to $(\mathring{\mathbf{n}}\cdot\mathbf{u}_{,1})(\mathring{\mathbf{a}}_1\cdot\mathbf{u}_{,1})$, that is $\overline{\mathcal{U}}_{\mathrm{m}}^{(2)\mathrm{B}}$, which can be written as

$$\begin{aligned}
\overline{\mathcal{U}}_{\mathrm{m}}^{(2)\mathrm{B}} &= -\frac{8(1-\nu^2)}{R^2}\lambda_{\mathrm{m}}\int_0^{\pi/2}\frac{\mathring{\rho}_{,\varphi}\,\mathring{z}_{,\varphi}}{(\mathring{\rho}_{,\varphi}^{\,2}+\mathring{z}_{,\varphi}^{\,2})^{5/2}}(\mathring{\mathbf{n}}\cdot\mathbf{u}_{,1})(\mathring{\mathbf{a}}_1\cdot\mathbf{u}_{,1})\,\mathring{a}\mathrm{d}\varphi \\
&= -\frac{8(1-\nu^2)}{R^2}\lambda_{\mathrm{m}}\int_0^{\pi/2}\frac{\mathring{\rho}_{,\varphi}\,\mathring{z}_{,\varphi}}{(\mathring{\rho}_{,\varphi}^{\,2}+\mathring{z}_{,\varphi}^{\,2})^{5/2}}\Big(w_{,1}+\mathring{b}^1_1 u_1\Big)\left[\Big(-\frac{(\mathring{\psi}(\varphi)\mathring{a})_{,1}}{\mathring{a}} + \frac{1}{2}\frac{\mathring{a}_{11,1}}{\mathring{a}_{11}}\mathring{\psi}(\varphi)\Big)u_1 - \mathring{b}_{11}w\right]\mathring{a}\mathrm{d}\varphi \\
&= -\frac{8(1-\nu^2)}{R^2}\int_0^{\pi/2}\mathbf{m}(\mathbf{u})\cdot\mathbf{q}\,\mathring{a}\mathrm{d}\varphi\,,
\end{aligned} \tag{49}$$

where $\mathbf{m}(\mathbf{u})$ is a distributed torque, which is a linear function of the displacement field, expressed as

$$\mathbf{m}(\mathbf{u}) = m^\alpha\mathring{\mathbf{a}}_\alpha = \lambda_{\mathrm{m}}\frac{\mathring{\rho}_{,\varphi}\,\mathring{z}_{,\varphi}}{(\mathring{\rho}_{,\varphi}^{\,2}+\mathring{z}_{,\varphi}^{\,2})^{3/2}}\left[\Big(-\frac{(\mathring{\psi}(\varphi)\mathring{a})_{,1}}{\mathring{a}} + \frac{1}{2}\frac{\mathring{a}_{11,1}}{\mathring{a}_{11}}\mathring{\psi}(\varphi)\Big)u_1 - \mathring{b}_{11}w\right]\mathring{\mathbf{a}}_1 = m^1\mathring{\mathbf{a}}_1\,, \quad \text{with} \quad m^2 = 0\,. \tag{50}$$

Therefore, the term $\overline{\mathcal{U}}_{\mathrm{m}}^{(2)\mathrm{B}}$ corresponds to a distribution of torques across the shell, which depend linearly on the displacement field. We will analyze the magnitude of the first and second-order terms studied in this section and show how the term $\overline{\mathcal{U}}_{\mathrm{m}}^{(2)\mathrm{A}}$, corresponding to a distribution of linear rotational springs, dominates over the other terms.

3.3 Scaling analysis

Now that we have a full interpretation of the magnetic energy – Supplementary Eqs. (41), (45) and (49) –, we perform a scaling analysis to understand how the buckling pressure depends on the magnetic field, the geometry of the shell and its material properties. We will proceed by balancing the potential associated to the live pressure and the magnetic energy.

First, we note that the undeformed surface coordinates, as well as their derivatives, scale as the characteristic radius of the shell; e.g., $\mathring{\rho} \sim R$ and $\mathring{\rho}_{,\varphi} \sim R$. Furthermore, as we are interested in the onset of buckling that takes place within the linear deformation regime, we assume that both the in-plane and normal displacements scale linearly with the shell thickness, $|\check{\mathbf{u}}| \sim h$ and $w \sim h$, all the way up to the onset of buckling.

Scaling of the first-order term, $\overline{\mathcal{U}}_{\mathbf{m}}^{(1)}$. Considering the above assumptions for the displacement field, we find the following scaling for the first-order term of the reduced magnetic energy in in Supplementary Eq. (41):

$$\overline{\mathcal{U}}_{\rm m}^{(1)} = \frac{8(1-\nu^2)}{R^2}\lambda_{\rm m}\int_0^{\pi/2} R^{-2}\Big[\mathring{\psi}(\varphi)\mathring{b}_{11}w - \Big(-\frac{(\mathring{\psi}(\varphi)\mathring{a})_{,1}}{\mathring{a}} + \frac{1}{2}\mathring{\psi}(\varphi)\frac{\mathring{a}_{11,1}}{\mathring{a}_{11}}\Big)u_1\Big]\,\mathring{a}\,\mathrm{d}\varphi \sim \lambda_{\rm m}\frac{h}{R}\,. \tag{51}$$

This scaling can be derived as follows. The first term within the square brackets scales as Rh, given that the function $\mathring{\psi}(\varphi)$ is dimensionless, whereas $\mathring{b}_{11} \sim R$ and $w \sim h$. The second term also scales as Rh, given that the term within the round brackets is dimensionless and $u_1 \sim Rh$ (the magnitude of the in-plane displacement field scales as h but the covariant components scale as Rh since the contravariant base vectors scale as $1/R$). By performing the required multiplications, remembering also that $\mathring{a} \sim R^2$, it follows that $\overline{\mathcal{U}}_{\rm m}^{(1)} \sim \lambda_{\rm m}h/R$.

Scaling of the second-order term, $\overline{\mathcal{U}}_{\mathbf{m}}^{(2)\mathrm{A}}$. We have to first focus on the scaling of the rotation vector $\mathbf{q}$ in Supplementary Eq. (43). Given that the majority of the deformation of the shell takes place in the neighborhood of the north pole, within a length that scales with the boundary layer $\sim \sqrt{Rh}$, angles will scale with the angular width of that boundary layer as $\sim \sqrt{h/R}$. This result means that $|\mathbf{q}| \sim \sqrt{h/R}$ or, equivalently, $\mathbf{q}\cdot\mathbf{q} \sim h/R$. Finally, recalling that the function $\mathring{\chi}(\varphi)$ is dimensionless, we can derive the scaling of $\overline{\mathcal{U}}_{\rm m}^{(2)\mathrm{A}}$ as

$$\overline{\mathcal{U}}_{\rm m}^{(2)\mathrm{A}} = \frac{8(1-\nu^2)}{R^2}\lambda_{\rm m}\int_0^{\pi/2}\frac{1}{2}\mathring{\chi}(\varphi)\mathbf{q}\cdot\mathbf{q}\,\mathring{a}\,\mathrm{d}\varphi \sim \lambda_{\rm m}\frac{h}{R}\,, \tag{52}$$

which scales exactly as the first-order term $\overline{\mathcal{U}}_{\rm m}^{(1)}$, in Supplementary Eq. (51) above.

Scaling of the second-order term, $\overline{\mathcal{U}}_{\mathbf{m}}^{(2)\mathrm{B}}$. The scaling for this term can be derived by applying the same arguments that we used for $\overline{\mathcal{U}}_{\rm m}^{(2)\mathrm{A}}$, for which we first turn to the second line of Supplementary Eq. (49). The first term of the multiplication scales as $1/R^3$. The second term, that is, the covariant component of the rotation vector, scales as $\sqrt{Rh}$ as we have seen previously. Finally, the third term in the square brackets scales as Rh, exactly as we have seen for the first-order energy term. Multiplying all these terms, we conclude that the second-order term $\overline{\mathcal{U}}_{\rm m}^{(2)\mathrm{B}}$ scales as

$$\overline{\mathcal{U}}_{\rm m}^{(2)\mathrm{B}} = -\frac{8(1-\nu^2)}{R^2}\int_0^{\pi/2}\mathbf{m}(\mathbf{u})\cdot\mathbf{q}\,\mathring{a}\,\mathrm{d}\varphi \sim \lambda_{\rm m}\Big(\frac{h}{R}\Big)^{\frac{3}{2}}\,. \tag{53}$$

Scaling of the potential associated to the live pressure, $\overline{\mathcal{U}}_p$. In the pre-buckling regime (during loading up to the buckling event), the displacement field is normal to the middle surface of the shell and scales with its thickness, differently from what happens in the post-buckling regime (after a dimple has formed). Consequently, in the pre-buckling regime, the change in volume scales as R^2h[17]. A more formal way to obtain this result is to compute the difference in volume assuming a normal displacement of order h so that the deformed radius is equal to $R-h$. In this case, purely from geometry, the change in volume is

$$\Delta V = \frac{4}{3}\pi(R-h)^3 - \frac{4}{3}\pi R^3 = 4\pi R^2 h\Big(1 + \frac{h}{R} + \frac{1}{3}\Big(\frac{h}{R}\Big)^2\Big) \sim R^2 h\,. \tag{54}$$

Given that we have chosen to nondimensionalize the volume by R^2h, the dimensionless change in volume will be of order 1. Hence, the live pressure potential scales as

$$\overline{\mathcal{U}}_{\rm p} = 8(1-\nu^2)\frac{p}{E}\Delta\overline{V} \sim \frac{p}{E}\,. \tag{55}$$

We highlight that the above scaling argument for the critical volume change, ΔV, is based on a uniformly compressed sphere, corresponding to deformations where a dimple has not yet formed. Indeed, right past the buckling event (i.e., when a dimple has formed), the analogous scaling to Supplementary Eq. (54) would be

$\Delta V \sim h^2 R$, given that the height of the dimple is assumed to be of the order of the thickness and its width scales as $\sqrt{Rh}$. This scaling argument is only valid immediately after buckling, that is, for unstable shapes, and cannot be used in general for the postbuckling regime as a whole, where the amplitude of the dimple becomes of the same order of shell radius.

Scaling law of the change of knockdown factor, $\Delta\kappa_{\rm d}$, with respect to the non-magnetic case. We can now balance the live pressure potential with the reduced magnetic energy to obtain the scaling for the change of knockdown factor due to the applied magnetic field. Combining the scaling results for the first and second-order terms of the magnetic energy, we conclude that

$$\overline{\mathcal{U}}_{\rm m} = \overline{\mathcal{U}}_{\rm m}^{(1)} + \overline{\mathcal{U}}_{\rm m}^{(2)\rm A} + \overline{\mathcal{U}}_{\rm m}^{(2)\rm B} \sim \lambda_{\rm m}\frac{h}{R}\,. \tag{56}$$

Recalling Eq. (1) of the main article, we note that the classic prediction for the buckling pressure of a perfect spherical shell scales as $p_c \sim E(h/R)^2$. Hence, we find that the knockdown factor (defined as $\kappa_{\rm d} = p_{\rm max}/p_{\rm c}$, where $p_{\rm max}$ actual critical buckling pressure of the imperfect shell) scales as $\kappa_{\rm d} \sim (p/E)(R/h)^2$. Consequently, the change of knockdown factor with respect to the non-magnetic case, $\kappa_{\rm d} = \kappa_{\rm d} - \kappa_{\rm d}(B^{\rm a} = 0)$, scales as $\Delta\kappa_{\rm d} \sim (\Delta p/E)(R/h)^2$. Balancing the scalings for the potential of Δp, $\overline{\mathcal{U}}_{\Delta p} = \Delta p/E$, and the magnetic energy in Supplementary Eq. (56) yields

$$\frac{\Delta p}{E} \sim \lambda_{\rm m}\frac{h}{R}\,. \tag{57}$$

Hence, the scaling of the change of knockdown factor is

$$\Delta\kappa_{\rm d} \sim \frac{\Delta p}{E}\left(\frac{R}{h}\right)^2 \sim \lambda_{\rm m}\frac{R}{h}\,, \tag{58}$$

From the result in Supplementary Eq. (58), we define the *magneto-elastic buckling parameter*,

$$\Lambda_{\rm m} = \lambda_{\rm m}\frac{R}{h}, \tag{59}$$

which governs the knockdown factor under combined pressure and magnetic loading of spherical magnetic shells. The scaling $\Delta\kappa_{\rm d} \sim \Lambda_{\rm m}$ allows us to rationalize how the magnetic field modifies the buckling pressure for shells with different radius-to-thickness ratios.

3.4 Quantification of the effect of the magnetic field on shell buckling

Towards gaining a better physical understanding of the magnitude of the first and second order terms of the reduced magnetic energy, we ran two separate sets of simulations for shells containing a defect of amplitude $\delta/h = 0.39$ and angular width $\overline{\varphi}_\circ = 3.2$. First, we used (i) the fully nonlinear magnetic energy ($\overline{\mathcal{U}}_{\rm m}$, Supplementary Eq. (18)). Second, we considered its terms at the different orders: (ii) up to second-order ($\overline{\mathcal{U}}_{\rm m}^{(1)} + \overline{\mathcal{U}}_{\rm m}^{(2)\rm A} + \overline{\mathcal{U}}_{\rm m}^{(2)\rm B}$, Supplementary Eqs. (41), (45), and (49), respectively), (iii) the first-order term only ($\overline{\mathcal{U}}_{\rm m}^{(1)}$, Supplementary Eq. (41)), and (iv) the second-order term relevant to the shell rotation only ($\overline{\mathcal{U}}_{\rm m}^{(2)\rm A}$, Supplementary Eq. (45)).

In Supplementary Fig. 5, we plot the loading paths of magnetic shells in both the plane $\bar{p} - \Delta\overline{V}$ (Supplementary Fig. 5**a**) and in the plane $\bar{p} - \overline{w}$ (Supplementary Fig. 5**b**), for three different values of the applied magnetic field $B^{\rm a} = \{66, 0, -66\}\,\rm mT$ (red, black, and blue lines, respectively). In both plots, the black curves correspond to the classic case of pressure buckling of the shell ($B^{\rm a} = 0\,\rm mT$)[2]. The solid curves correspond to the results from the fully nonlinear magnetic energy $\overline{\mathcal{U}}_{\rm m}$. When the magnetic field is applied, the loading paths are modified with respect to the zero field case; there is a marked difference between the red/blue curves and the black curves. Consequently, there is a finite change of the knockdown factor, as reported in detail in Figs. 3 and 4 of the main article. The dotted lines correspond to the loading paths obtained considering only the linear term of the reduced magnetic energy; clearly showing that the linear term alone is unable to reproduce the observed finite change in knockdown factor. By contrast, we find that the second-order magnetic energy term $\overline{\mathcal{U}}_{\rm m}^{(2)\rm A}$ is able to accurately reproduce the fully nonlinear behavior; the dotted-dashed curves are close to the solid curves, especially in the pre-buckling regime, up to the onset of the instability. Therefore, the second-order term in the magnetic energy, which includes the contribution of the distributed magnetic torque relevant to the shell rotation, dominates the modification of the critical buckling conditions due to the applied magnetic field.

Distribution of the magnetic torque and its dependence on the applied pressure. The magnetic torque, $\boldsymbol{\tau} = -k(\varphi)\mathbf{q}$, is non-uniformly distributed along the shell, depending on the rotation vector, $\mathbf{q}$, and the equivalent rotational stiffness, $k(\varphi) = \lambda_{\rm m}\mathring{\chi}(\varphi)$ (Supplementary Eq. (46)). Here, we want to understand how the magnetic torque varies as a function of the rotation vector, for positive and negative applied magnetic fields,

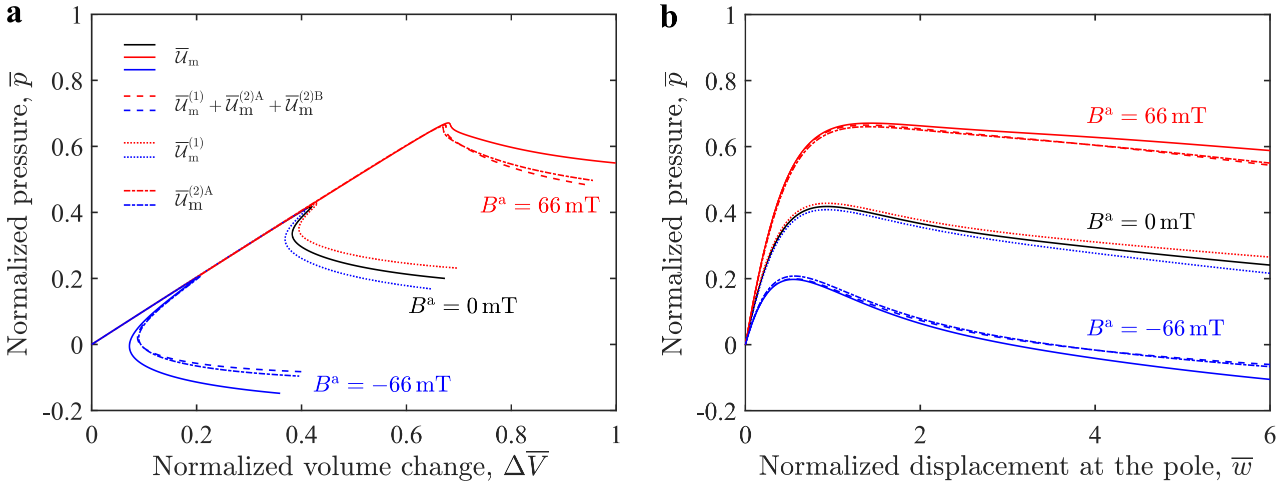

Supplementary Fig. 5: Load-carrying behavior predictions for a magnetic spherical shell from our 1D model. Different terms of magnetic energy were considered in the model: (i) the fully nonlinear magnetic energy ($\overline{\mathcal{U}}_{\mathrm{m}}$, solid lines), (ii) the magnetic energy for small displacements up to second-order ($\overline{\mathcal{U}}_{\mathrm{m}}^{(1)}+\overline{\mathcal{U}}_{\mathrm{m}}^{(2)\mathrm{A}}+\overline{\mathcal{U}}_{\mathrm{m}}^{(2)\mathrm{B}}$, dashed lines), (iii) the first-order term only ($\overline{\mathcal{U}}_{\mathrm{m}}^{(1)}$, dotted lines), and (iv) the second-order term relevant to the shell rotation only ($\overline{\mathcal{U}}_{\mathrm{m}}^{(2)\mathrm{A}}$, dash-dotted lines). The results are presented for different values of the external magnetic flux density $B^{\mathrm{a}}=\{66,0,-66\}\,\mathrm{mT}$, represented by the red, black, and blue curves, respectively. The shell contains a defect of amplitude $\delta/h=0.39$ and width $\overline{\varphi}_{\circ}=3.2$, which corresponds to the experiments in Fig. 1**d** of the main article. (a) Normalized pressure, $\overline{p}=p/p_{\mathrm{c}}$, as a function of normalized volume change, $\Delta\overline{V}=\Delta V/\Delta V_{\mathrm{c}}$, of the shell. Buckling occurs at the maximum value, which defines the knockdown factor. (b) Normalized pressure, $\overline{p}$, as a function of normalized displacement at the pole, $\overline{w}=w/h$.

so as to shed light on the mechanism that underlies the change in knockdown factor. To do so, we selected the representative shell containing a defect of amplitude $\overline{\delta}=0.39$ and width $\overline{\varphi}_{\circ}=3.2$, which was tested in the experiments (Fig. 1**d** of the main article).

In Supplementary Fig. 6, we plot the predictions from our model for the magnitudes of the rotation vector, $|\mathbf{q}|=\sqrt{\mathring{a}^{11}}q_1$, and the torque vector, $|\boldsymbol{\tau}|=\sqrt{\mathring{a}_{11}}\tau^1$, along the profile of the shell. Apart from the case of zero magnetic field, two representative values of the magnetic field were considered: $B^{\mathrm{a}}=66\,\mathrm{mT}$ (Supplementary Fig. 6**a**) and $B^{\mathrm{a}}=-66\,\mathrm{mT}$ (Supplementary Fig. 6**b**). For each case, we present results for the four pressure levels represented by the open circles marked on the loading curves in Supplementary Fig. 6**c**. We find that the shell rotation is localized both at the defect (i.e., at the pole of the shell, $\varphi=0$) and near the equator. Due to the non-uniformity of the equivalent rotational stiffness, k, the torque is prominent at the pole but almost zero in the rest of the shell. The magnetic torque increases with the rotation during pressurization.

Note that, for the case when the field is parallel to the shell magnetization ($B^{\mathrm{a}}=66\,\mathrm{mT}$), the directions of rotation and torque are opposite. By contrast, for the case when the field is anti-parallel (opposite) to the shell magnetization ($B^{\mathrm{a}}=-66\,\mathrm{mT}$), the rotation and torque are in the same direction. We have integrated the distributed torque on the shell middle surface and, in Supplementary Fig. 6**d**, we plot the normalized resultant torque, $\tau_{\mathrm{r}}=\frac{1}{Rh}\iint\sqrt{\mathring{a}_{11}}\tau^1\ \mathring{a}\mathrm{d}\theta\mathrm{d}\varphi$, as a function of pressure. Note that the initial value of τ_{r} at $p/p_{\mathrm{c}}=0$ has been subtracted. This initial value represents the total torque in the pressure-free state and was verified to have a negligible effect on the critical loads. Interestingly, we find that the torque does not increase linearly with pressure, with a slope that increases and becomes remarkably steep as the pressure approaches the critical value ($\overline{p}_{\mathrm{max}}$ represented by the dash lines in Supplementary Fig. 6**d**), where large rotation occurs.

In conclusion, we find that the change of the knockdown factor is dominantly induced by the torque localized near the defect, while the torque is almost zero throughout the rest of the shell, where rotations are negligible. As such, one should be able to extend the proposed approach, which has been demonstrated on hemispherical shells, for full spherical shells and even shallow shell sections, guaranteed by the localized nature of shell buckling. This would be an extension of classical methods in shell mechanics where, given the shallowness of the buckling mode, studies on the buckling of shallow spherical shells[18] have proven to be successful in predicting the buckling of the corresponding full spherical shells[14].

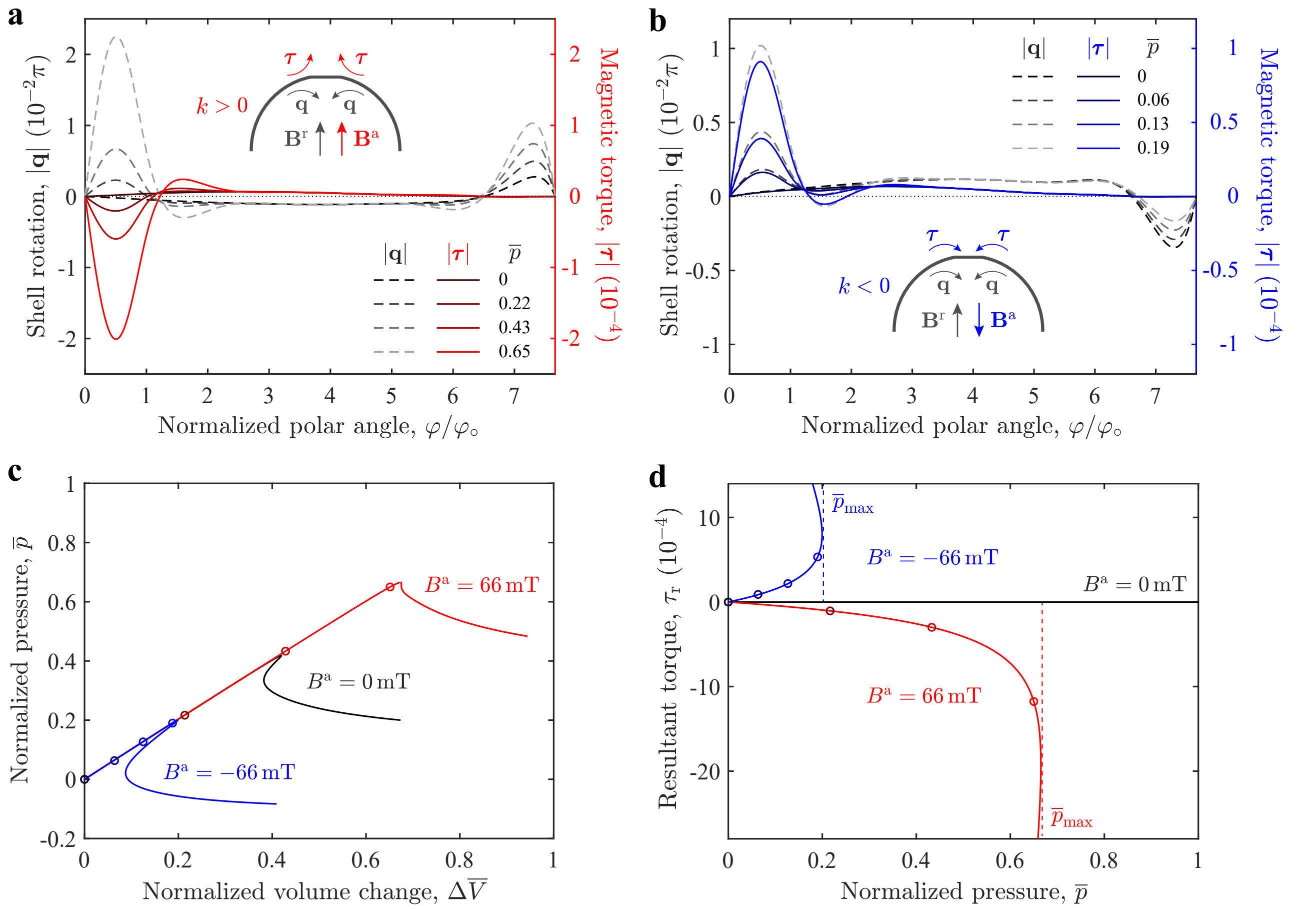

Supplementary Fig. 6: Shell rotation and magnetic torque predicted by our theoretical model. Distributions of shell rotation $|\mathbf{q}| = \sqrt{\mathring{a}^{11}}q_1$ (magnitude of $\mathbf{q}$) and of the associated magnetic torque $|\boldsymbol{\tau}| = \sqrt{\mathring{a}_{11}}\tau^1$ (magnitude of $\boldsymbol{\tau}$) along the shell 2D profile for $B^{\mathrm{a}} = 66\,\mathrm{mT}$ (**a**) and $B^{\mathrm{a}} = -66\,\mathrm{mT}$ (**b**). The computations were carried out on the shell containing a defect of amplitude $\overline{\delta} = 0.39$ and width $\overline{\varphi}_{\circ} = 3.2$, which corresponds to the experiments in Fig. 1**d** of the main article. Terms up to second-order, $\overline{\mathcal{U}}^{(1)}_{\mathrm{m}} + \overline{\mathcal{U}}^{(2)\mathrm{A}}_{\mathrm{m}} + \overline{\mathcal{U}}^{(2)\mathrm{B}}_{\mathrm{m}}$, were considered for the magnetic energy. The results are presented at different values of the pressure level (see legend), corresponding to the open circles on the loading curves $\overline{p}(\Delta\overline{V})$ in **c** and $\tau_{\mathrm{r}}(\overline{p})$ curves in **d**. **d**, Normalized resultant magnetic torque imposed in the shell, τ_{r}, versus normalized pressure $\overline{p}$, corresponding to the three loading cases in **c**. The critical values, $\overline{p}_{\mathrm{max}}$, corresponds to the peaks of $\overline{p}(\Delta\overline{V})$ curves in **c**.

Supplementary Note 4 Effect of the Shell Geometry on Buckling

In this note, we report the results from a parametric study where we have systematically varied the following two parameters: the shell radius-to-thickness ratio, R/h, and the defect width, $\overline{\varphi}_{\circ}$. We set out to investigate the effect of the shell geometry on buckling of magnetic shells. In Section 4.1, we explored R/h over a wider range ($50 \leq R/h \leq 400$) than that of the experiments ($R/h = 79.1$ for MRE-22 shells and $R/h = 91.3$ for MRE-32 shells), so as to more unequivocally establish the independence of R/h on both the knockdown factor and its change under the applied magnetic field. The effect of the defect width on the critical buckling conditions is also investigated in Section 4.2.

4.1 Independence of shell radius-to-thickness ratio

Using the 1D model for magnetic shells that we presented in Supplementary Note 3, we obtained numerical solutions for a series of shells with different values of the radius-to-thickness ratio $R/h = \{50, 100, 200, 400\}$, and quantified the predictions for the knockdown factor, κ_{d}, as well as its change with respect to the non-magnetic case, $\Delta\kappa_{\mathrm{d}}$, of the shell. The results are shown in Supplementary Fig. 7 for different values of the magneto-elastic buckling parameter $\Lambda_{\mathrm{m}} = \{0.18, 0.09, 0, -0.09, -0.18\}$. We recall from the definition in Supplementary Eq. (59) that $\Lambda_{\mathrm{m}} = \lambda_{\mathrm{m}}R/h$. We considered defects with increasing amplitudes $0.1 \leq \overline{\delta} \leq 4$, while fixing the defect width at $\overline{\varphi}_{\circ} = 3.2$. We find that, for any given defect geometry, both κ_{d} and $\Delta\kappa_{\mathrm{d}}$ are governed by the parameter Λ_{m}, independently of R/h (there is a near-collapse of the $\kappa_{\mathrm{d}}(\overline{\delta})$ curves for the different R/h values).

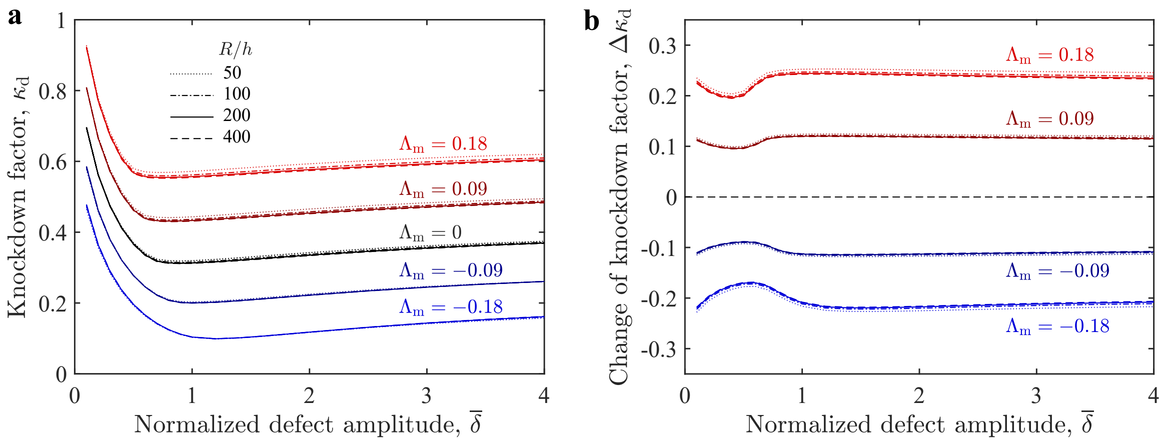

Supplementary Fig. 7: Independence of shell radius-to-thickness ratio. **a**, Knockdown factor, κ_d, and **b**, its change $\Delta\kappa_d$ with respect to the non-magnetic case versus the amplitude of the defect, $\bar{\delta}$. The various values of the radius-to-thickness ratio are represented by the different line types: $R/h = 50$ (dotted lines), $R/h = 100$ (dash-dotted lines), $R/h = 200$ (solid lines), and $R/h = 400$ (dashed lines). The defect width is fixed at $\bar{\varphi}_\circ = 3.2$.

4.2 Effect of width of the defect on the change of knockdown factor

We also investigated the effect of the defect width, $\bar{\varphi}_\circ$, on the critical buckling load using our magnetic shell model. We explored the following values $\bar{\varphi}_\circ = \{1.0, 2.0, 3.0, 4.0\}$ and computed the prediction for both the knockdown factor, κ_d, of the shell and its change with respect to the non-magnetic case, $\Delta\kappa_d$, for different magnetic loading conditions. In Supplementary Fig. 8**a**, we present the predicted $\kappa_d(\bar{\delta})$ curves as a function of defect amplitude, $\bar{\delta}$, in the absence of the magnetic field ($\Lambda_m = 0$). We find that κ_d decreases for wider defects (i.e., increasing $\bar{\varphi}_\circ$). Then, we imposed the magnetic loading and varied the magnetic flux density, which is included in the magneto-elastic buckling parameter according to Supplementary Eq. (58), by setting the values: $\Lambda_m = \{0.18, 0.09, 0, -0.09, -0.18\}$. The associated change of knockdown factor with respect to the non-magnetic case, $\Delta\kappa_d$, is plotted in Supplementary Fig. 8**b**. We find that, when the defect amplitude is larger than a given threshold (determined by the differentiation of $\Delta\kappa_d$ with respect to $\bar{\delta}$ smaller than 1% and represented by the symbol on each curve of the plot) the change of knockdown factor becomes independent of the defect width (the curves for the various values of $\bar{\varphi}_\circ$ collapse). For relatively small defects ($\bar{\delta}$ below the threshold value), $\Delta\kappa_d$ depends, but only slightly, on $\bar{\varphi}_\circ$. The corresponding variation is lower than 8% for the extreme case explored of $\bar{\varphi}_\circ = 4.0$ and $\Lambda_m = -0.18$; less sensitively in comparison with κ_d, which decreases by up to 45% for the same parameter set.

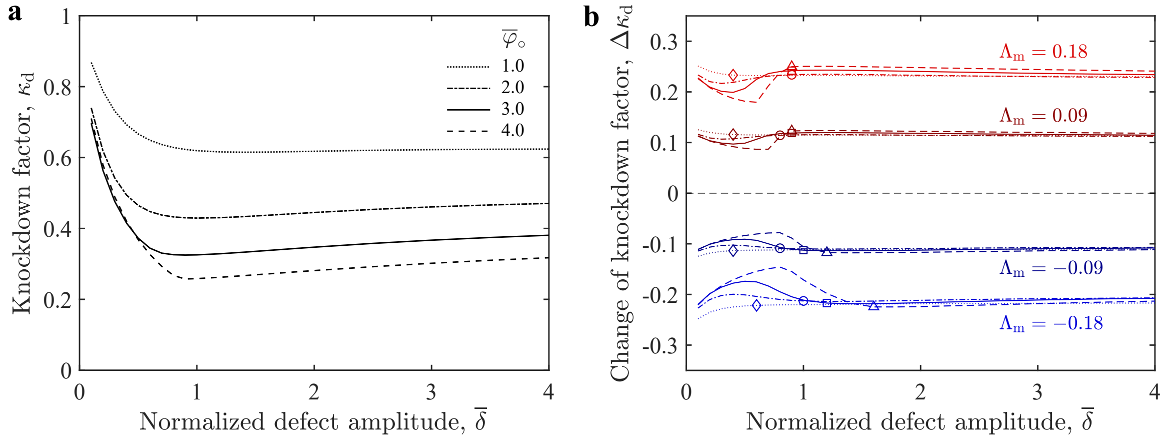

Supplementary Fig. 8: Effect of defect width. **a**, Knockdown factor, κ_d, versus defect amplitude, $\bar{\delta}$, for shells with different defect widths $\bar{\varphi}_\circ = \{1.0, 2.0, 3.0, 4.0\}$ (as detailed in the legend), pressurized in the absence of the magnetic field. **b**, Change of knockdown factor, $\Delta\kappa_d$, when imposing the magnetic field, versus defect amplitude, $\bar{\delta}$, for shells with different defect widths: $\bar{\varphi}_\circ = 1.0$ (dotted lines), $\bar{\varphi}_\circ = 2.0$ (dash-dotted lines), $\bar{\varphi}_\circ = 3.0$ (solid lines), and $\bar{\varphi}_\circ = 4.0$ (dashed lines). The external flux density is varied by $\Lambda_m = \{0.18, 0.09, 0, -0.09, -0.18\}$. The shell radius-to-thickness ratio is chosen to be $R/h = 200$.

References

1. Lee, A. *et al.* Fabrication of slender elastic shells by the coating of curved surfaces. *Nat. Commun.* **7**, 11155 (2016).

2. Lee, A., López Jiménez, F., Marthelot, J., Hutchinson, J. W. & Reis, P. M. The geometric role of precisely engineered imperfections on the critical buckling load of spherical elastic shells. *J. Appl. Mech.* **83**, 111005 (2016).

3. Timoshenko, S. P. & Woinowsky-Krieger, S. *Theory of Plates and Shells* (McGraw-Hill, 1959).

4. Kim, Y., Yuk, H., Zhao, R., Chester, S. A. & Zhao, X. Printing ferromagnetic domains for untethered fast-transforming soft materials. *Nature* **558**, 274 (2018).

5. Zhao, R., Kim, Y., Chester, S. A., Sharma, P. & Zhao, X. Mechanics of hard-magnetic soft materials. *J. Mech. Phys. Solids* **124**, 244–263 (2019).

6. Bertotti, G. *Hysteresis in Magnetism: for Physicists, Materials Scientists, and Engineers* (Academic Press, 1998).

7. Niordson, F. I. *Shell Theory.* North-Holland Series in Applied Mathematics and Mechanics (Elsevier Science, 1985).

8. Ciarlet, P. *Theory of Shells.* Mathematical Elasticity (Elsevier Science, 2000).

9. Audoly, B. & Pomeau, Y. *Elasticity and Geometry: from Hair Curls to the Non-linear Response of Shells* (Oxford University Press, 2010).

10. Pezzulla, M. & Reis, P. M. A weak form implementation of nonlinear axisymmetric shell equations with examples. *J. Appl. Mech.* **84**, 034501 (2019).

11. Gurtin, M. E., Fried, E. & Anand, L. *The Mechanics and Thermodynamics of Continua* (Cambridge University Press, 2010).

12. do Carmo, M. *Differential Geometry of Curves and Surfaces: Revised and Updated Second Edition.* Dover Books on Mathematics (Dover Publications, 2016).

13. O'Neill, B. *Elementary Differential Geometry* (Academic Press, 1997).

14. Koiter, W. T. The nonlinear buckling behavior of a complete spherical shell under uniform external pressure, parts i, ii, iii & iv. *Proc. Kon. Ned. Ak. Wet.* **B72**, 40–123 (1969).

15. Hutchinson, J. W. Buckling of spherical shells revisited. *Proc. R. Soc. A* **472**, 20160577 (2016).

16. Pezzulla, M., Stoop, N., Steranka, M. P., Bade, A. J. & Holmes, D. P. Curvature-induced instabilities of shells. *Phys. Rev. Lett.* **120**, 048002 (2018).

17. Hutchinson, J. W. & Thompson, J. M. T. Nonlinear buckling behaviour of spherical shells: barriers and symmetry-breaking dimples. *Phil. Trans. R. Soc. A* **375**, 20160154 (2017).

18. Hutchinson, J. W. Imperfection sensitivity of externally pressurized spherical shells. *J. Appl. Mech.* **34**, 49–55 (1967).
